# Physics-inspired feature engineering and explainable machine learning for tensile strength prediction of CNT–Si₃N₄ reinforced FWTPET joints

**DOI:** 10.3389/frai.2026.1952539

**Published:** 2026-10-20

**Authors:** Krishnan Venkatachalam, Senthil Kumaran Selvaraj, R. Lokeshkumar, G. Sundaramali

**Affiliations:** 1 School of Computer Science and Engineering, Vellore Institute of Technology, Vellore, India; 2 School of Mechanical Engineering, Vellore Institute of Technology, Vellore, India

**Keywords:** composite materials processing, explainable artificial intelligence, feature engineering, physics inspired machine learning, tensile strength prediction

## Abstract

**Introduction:**

The prediction of the tensile strength of carbon nanotube (CNT)–silicon nitride (Si₃N₄) reinforced friction welding of tube-to-tube plate using an external tool (FWTPET) joints is crucial for improving joint performance.

**Methods:**

A physics-inspired machine learning framework was developed using 25 experimentally derived observations to investigate the relationships between welding parameters, engineered process descriptors, and tensile strength. Physically motivated descriptors were constructed to represent thermal input, reinforcement interaction, material flow, and dispersion effects. Hyperparameter optimization was performed for multiple regression models to improve predictive performance.

**Results:**

The XGBoost model showed the best performance, with an MAE of 5.70, an RMSE of 7.74, and an R² of 0.9772. SHAP analysis identified CNT content and the dispersion-related descriptor as the most influential predictors. Ablation analysis showed that the combination of the original experimental process variables provided greater predictability than the engineered variables alone or their combination, while the physics-inspired process variables provided further physically interpretable representations of the welding process.

**Discussion:**

The results indicate that small-data machine learning, domain-based feature engineering, and explainable analysis are potential techniques for predicting the tensile strength of FWTPET joints.

## 1 Introduction

In the era of modern engineering, high performance composite material has been given a lot of attention because of its remarkable mechanical properties, low weight, and improved structural reliability. They are widely used in the aerospace, automotive, marine, and structural engineering industries, where maximizing strength, durability, and weight reduction is essential. The use of advanced reinforcement materials like CNTs and ceramic particles has proven to be an effective method to improve the mechanical and tribological properties of composites (Senthil Kumaran et al., 2020; Senthil Kumaran, 2013). The reinforcement results in enhanced tensile strength, hardness, abrasion resistance, thermal stability, and load-bearing capacity and thus improves the application of composite materials in harsh working conditions (Iftikhar et al., 2021; Jagannatham et al., 2020; Liu et al., 2024). The mechanical properties of such composites are, however, very sensitive to processing, and the interaction of constituents is complicated, making accurate prediction and optimization of their properties difficult (Vimalan et al., 2020; Soto-Diaz et al., 2025; Siddhardh et al., 2020).

Traditional experimental techniques for the optimization of process parameters are usually time-consuming, resource consuming and economically costly. To fabricate and characterize composite materials, extensive experimental work must be carried out in the laboratory, which is also very challenging for the case of process variables and reinforcement combinations. Furthermore, experimental materials science data sets are generally small owing to the expensive and time-consuming nature of the specimen preparation, mechanical testing, and microstructural characterization (Jin et al., 2023; Venkatachalam et al., 2026). Therefore, it is difficult to use traditional statistical analysis to determine the nonlinear relationship between the mechanical properties of the process and the process parameters. In the past few years, machine learning techniques have been increasingly applied to model material behaviors such as complex and nonlinear ones and to make accurate predictions of mechanical properties from experimental data (Arif et al., 2025; Jatavallabhula et al., 2025). Machine learning has also been applied for prediction and optimization of friction welding and friction stir welding processes (Krishna and Mathivanan, 2026; Dorbane et al., 2025). Recent studies have further demonstrated the application of machine learning for predicting mechanical properties of friction stir welded joints (Elsheikh, 2023; Verma et al., 2018; Mothilal and Kumar, 2025). In the meantime, traditional machine learning models are often black-box systems and do not necessarily represent the physical mechanisms that control material properties (Lundberg and Lee, 2017; Zhang et al., 2026).

The paradigm of physics inspired machine learning has proved to be a promising one that combines both domain-specific knowledge and data-driven predictive modeling (Rahman et al., 2025; Watson et al., 2024). Physics inspired models have the capability of improving predictive ability and scientific interpretation by incorporating physically meaningful descriptors that are extracted from the processes and material interactions (Rooney et al., 2026; Faegh et al., 2025; Hao et al., 2022). The efficient representation of phenomena like heat generation, material flow behavior, reinforcement dispersion, and plastic deformation during processing is possible using feature engineering based on established principles in materials science that enables machine learning algorithms to model and describe the phenomena. Thus, the models can make accurate predictions, and they can also offer some insight into the physical mechanisms that affect mechanical performance (Rahman et al., 2025; Faegh et al., 2025; Mishra et al., 2021).

At the same time, Explainable Artificial Intelligence (XAI) has emerged as a crucial concept in enhancing the transparency and trustworthiness of machine learning models (Lundberg and Lee, 2017; Zhang et al., 2026). Explainability methods allow researchers to measure the contribution of specific features to model predictions and provide meaningful relationships between the process parameters and the material properties (Venkatachalam et al., 2026). These interpretations are particularly useful in material sciences, where understanding the impact of specific processing conditions is essential for both scientific validation and the optimization of processes. By combining XAI with physics inspired feature engineering, one can gain a deeper understanding of the change of tensile strength, which helps in making informed decisions in designing composite materials (Venkatachalam et al., 2026; Huo, 2026).

Another important challenge that exists with experimental materials datasets is often the small number of samples associated with the dataset, which requires the use of powerful validation strategies. Small data sets may not be suitable for traditional train-test split approaches, which can result in biased performance estimates and waste of valuable experimental data (Jin et al., 2023; Luo et al., 2025). Leave-One-Out Cross-Validation (LOOCV) achieves a reliable approach to large-scale modeling by utilizing all available samples without compromising the model’s evaluation. LOOCV is a statistically sound technique for performance estimation in small-scale experimental studies by using one of the samples for testing and the rest for training (Venkatachalam et al., 2026; Huo, 2026). LOOCV can be used in combination with more sophisticated machine learning algorithms and automated hyperparameter optimization techniques, which make predictive models derived from a small number of experimental observations more reliable and generalizable (Arif et al., 2025; Dorbane et al., 2025; Venkatachalam et al., 2026).

The goal of this research is to propose a complete physics inspired machine learning framework to predict a material property, the tensile strength, using experimental data of the composite material. The proposed framework leverages physics-based feature engineering, feature validation and selection techniques, multiple state-of-the-art machine learning models, Optuna-based hyperparameter optimization, and XAI techniques to provide a strong and explainable predictive pipeline. Moreover, LOOCV provides reliable performance assessment for small-sized experimental datasets, and includes residual and mechanical interpretation analysis, which gives scientific relevance to the predictive models. The proposed methodology not only allows for the prediction of the tensile strength but also gives the meaning of the relationships between process parameters, material interactions and mechanical performance. The results of this work would help advance the design and optimization of advanced composite materials using interpretable, data-efficient and science-based machine learning models.

For the past several years, machine learning on small experimental datasets has gained prominence as an important field of study in materials science, where obtaining experimental data is often constrained by the manufacturing price of materials, testing time, and the availability of certain materials and devices (Luo et al., 2025; Huo, 2026). Relatively speaking, the development of accurate prediction models in such scenarios calls for the implementation of proper validation methods as well as the choice of proper engineering features and algorithms (Jin et al., 2023; Venkatachalam et al., 2026). Currently, it has been proven that data-centric models can discover significant patterns and relations between different variables using only small amounts of experimental data (Luo et al., 2025; Huo, 2026). With these observations in mind, this work aims to utilize physics-driven feature engineering and leave-one-out cross-validation for the development of reliable prediction models based on small datasets.

Recent research has proven the use of machine learning in the field of prediction, optimization and process–property analysis in friction based welding. The different speeds of tools can affect the microstructural and mechanical properties of dissimilar welded joints, as revealed by investigations conducted on FWTPET, which shows that the process parameters have an impact on weld performance (Sabu et al., 2025). At the same time, machine learning models including Random Forest, XGBoost, and multilayer perceptron are also applied successfully to optimize friction stir welding of aluminum alloys (Myśliwiec et al., 2024). Machine learning has also been shown to be capable of predicting optimal parameter values and tensile strength for friction stir welding (Luesak et al., 2026) and predicting fracture load and ductility for Friction Stir Spot Welding (FSSW) (Sardar et al., 2026) in recent studies. In addition, machine learning has been used to predict the performance of FSSW of aluminum alloys, showing its potential for various material combinations and welding processes (Keshavamurthy et al., 2026). Another ensemble learning method has also been found to have satisfactory performance in predicting the ultimate tensile strength of asymmetric friction stir welding (Matitopanum et al., 2023) and weld properties of AA7075 friction stir welded joints (Matitopanum et al., 2023). In general, these studies demonstrate the promise of machine learning in modeling nonlinear welding process/property relationships, but the inclusion of domain-informed feature engineering and explainable machine learning techniques to model the CNT–Si₃N₄ reinforced FWTPET is still relatively under-researched and is a motivation for the present research.

## 2 Materials and methods

The process used in this research to predict CNT − Si₃N₄ reinforced FWTPET joints has been discussed (Siddhardh et al., 2020). These processes include selection of materials, specifications of tool and reinforcement, experimental design using Taguchi L25 orthogonal array (Siddhardh et al., 2020).

### 2.1 Tool material and design

High speed steel (HSS) tools are used for friction welding (FW) of AA6061 tube plate and AA6063 tube. Because the tool material needs to be sufficiently hard to weld the aluminum, it must be carefully chosen. Since HSS can tolerate high temperatures, it is favored in many equipment projects. In comparison to AA6063 (melting point 655 °C) and AA6061 (melting point 652 °C), the tool can withstand higher temperatures. The tool’s design is based on the tube’s ID. With a diameter of 15.4 mm and a guiding pin of 10 mm, the tool shoulder measures 35 mm in diameter. The purpose of the procedures, including turning and facing, is to create a polished and smooth shape (Siddhardh et al., 2020).

For the FWTPET studies, aluminum alloys AA6061 and AA6063 were chosen as the starting materials. Because of its superior weldability, resistance to corrosion, and advantageous strength-to-weight ratio, AA6063 was utilized as tube material and AA6061 as tube plate material. Table 1 shows the chemical composition of both alloys. The mechanical qualities and weld characteristics of the joints are affected by the variation in alloying elements, especially magnesium (Mg), silicon (Si), and copper (Cu). The chemical composition displayed in Table 1 was developed using these materials as the parent alloys for all experimental trials.

**Table 1:** Chemical composition of AA6061 and AA6063 aluminum alloys.

Element	AA6061 (wt.%)	AA6063 (wt.%)
Al	Remainder	Remainder
Si	0.40–0.80	0.20–0.60
Fe	0.70	0.35
Cu	0.15–0.40	0.10
Mn	0.15	0.10
Mg	0.80–1.20	0.45–0.90
Cr	0.04–0.35	0.10
Zn	0.25	0.10
Ti	0.15	0.10
Other (each)	0.05	0.05
Other (total)	0.15	0.15

### 2.2 Flat plate with tube having holes at the circumference

The tube plate was constructed from a 50 mm x 50 mm × 6.3 mm AA6061 aluminum alloy sheet. A hole measuring 19.4 mm in diameter was fabricated near the center of the plate to fit the AA6063 tube. To enable the external incorporation of reinforcing particles, 4 mm diameter apertures were created around the center opening along a pitch circle diameter. The configuration of the holes was crafted to keep all reinforcing voids within the shoulder contact zone of the tool, hence facilitating efficient material blending during the FWTPET procedure.

Figure 1 (Siddhardh et al., 2020) depicts the geometric measurements of the tube plate, encompassing the center aperture and the peripheral reinforcing openings. Figures 2a,b (Siddhardh et al., 2020) illustrate the constructed tube plate with the inserted tube prior to the welding process, emphasizing the positions of the reinforcement apertures designated for the incorporation of CNT and Si₃N₄ particles.

**Figure 1:**
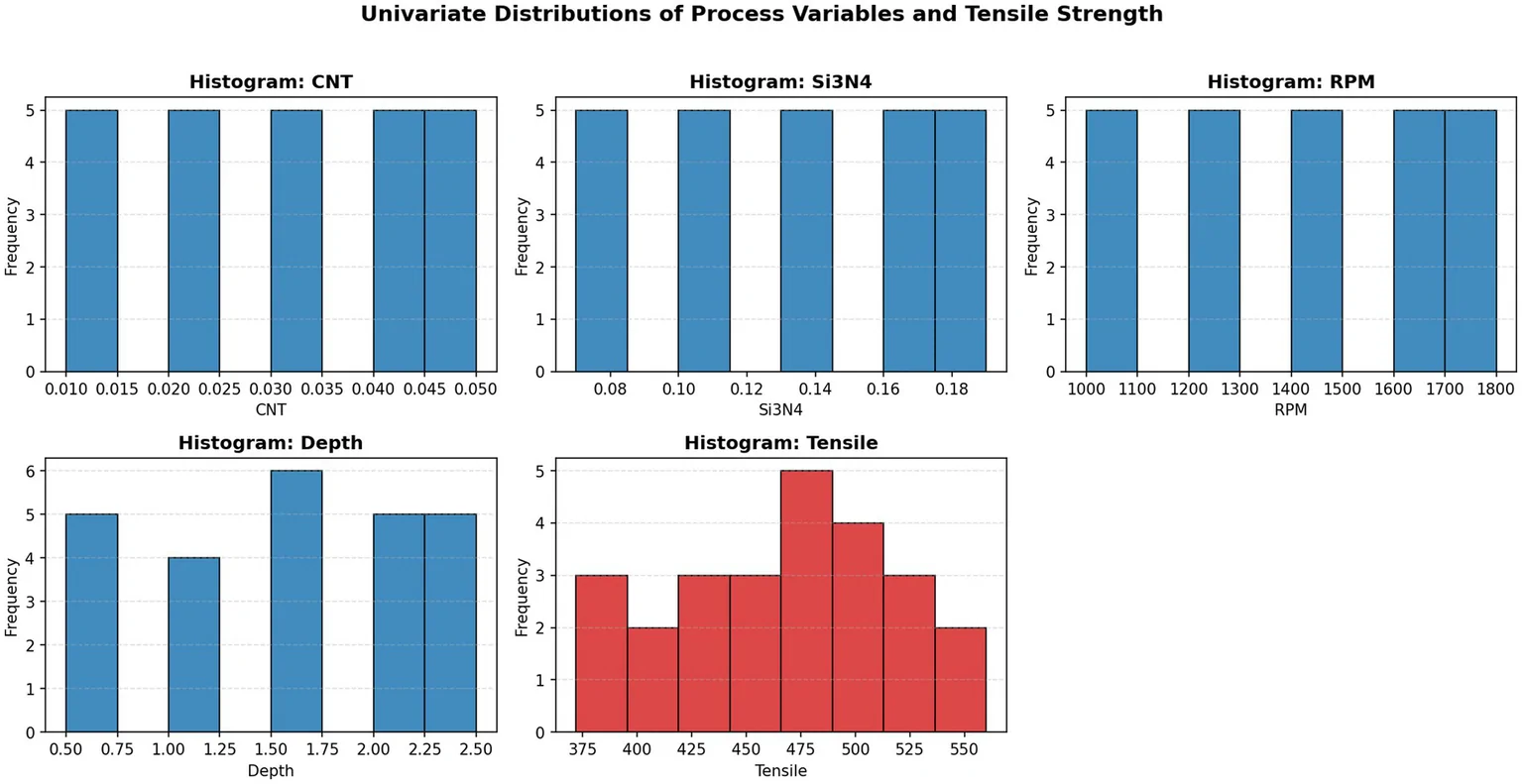
Univariate distributions of the process variables and tensile strength.

**Figure 2:**
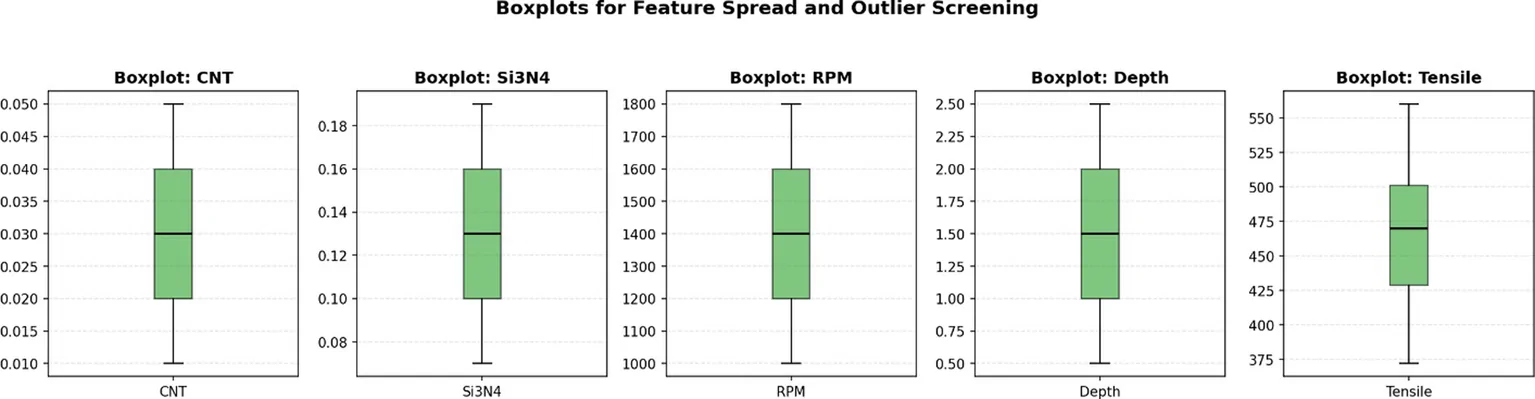
Boxplots for feature spread and outlier screening of the experimental dataset.

### 2.3 Selection of reinforcements

CNTs and Si₃N₄ particles were used as reinforcement materials to improve the mechanical properties of the friction-welded connections. Multiwalled carbon nanotubes (MWCNTs) exhibit remarkable tensile strength and high elasticity, and superior thermal conductivity, rendering them efficient reinforcing agents for enhancing load distribution and grain refinement in the weld zone. Si₃N₄, a superior ceramic substance, demonstrates remarkable hardness, abrasion resistance, thermal stability, and chemical inertness. The integration of CNT and Si₃N₄ reinforcements is anticipated to enhance the tensile strength and mechanical integrity of welded joints by facilitating superior reinforcement distribution and strengthening processes throughout the FWTPET procedure. The characteristics of the CNT and Si₃N₄ powders utilized in this research are encapsulated in Tables 2, 3.

**Table 2:** Specifications of multiwalled carbon nanotubes (CNTs).

Property	Specification
Type	Multiwalled Carbon Nanotubes (MWCNTs)
Color	Black Powder
Purity	>99%
Average Diameter	10–15 nm
Average Length	~5 μm
Amorphous Carbon	<1%
Surface Area	~400 m^2^/g

**Table 3:** Specifications of silicon nitride (Si₃N₄) reinforcement.

Property	Specification
Purity	99%
Average Particle Size (APS)	40–50 μm
Molecular Formula	Si₃N₄
Molecular Weight	140.28 g/mol
Form	Powder
Color	White
Density	2.2–3.5 g/cm^3^
Melting Point	1900 °C

### 2.4 Backing block design

In the FWTPET procedure illustrated in Figures 3 and 4a,b (Siddhardh et al., 2020), the backing block serves to guarantee an accurate alignment of the workpiece within the vertical milling machine. It aids the workpiece, guarantees a flawless union, prevents the tube from dislodging from its position, and halts the bending of the tube plate. This support block is constructed from mild steel, precisely measuring 50*50*50 mm, and features a drilled hole with a depth of 34.7 mm and a diameter of 19.4 mm. A space is allocated to guarantee a clearance fit.

**Figure 3:**
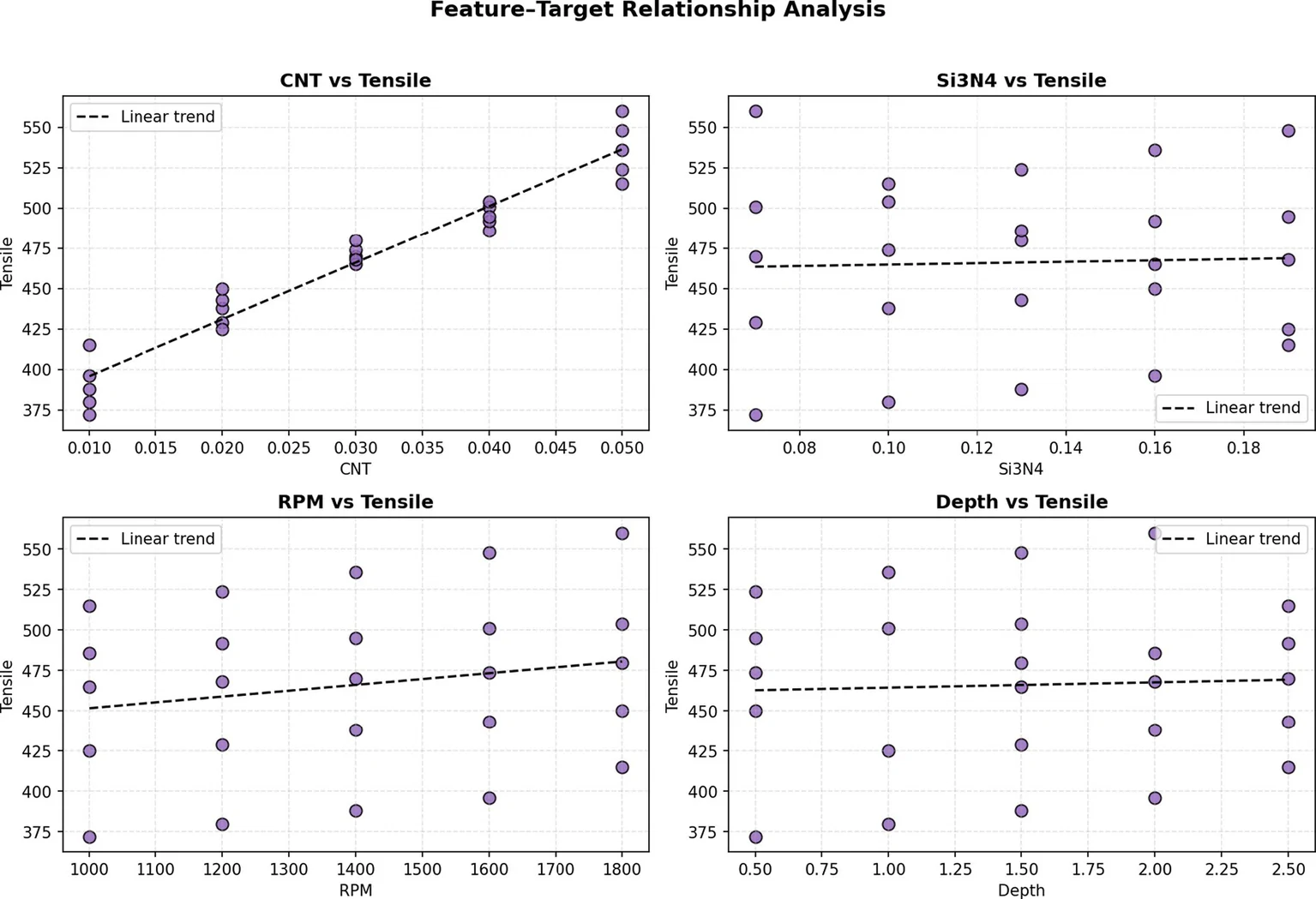
Feature–target relationship analysis between process variables and tensile strength.

**Figure 4:**
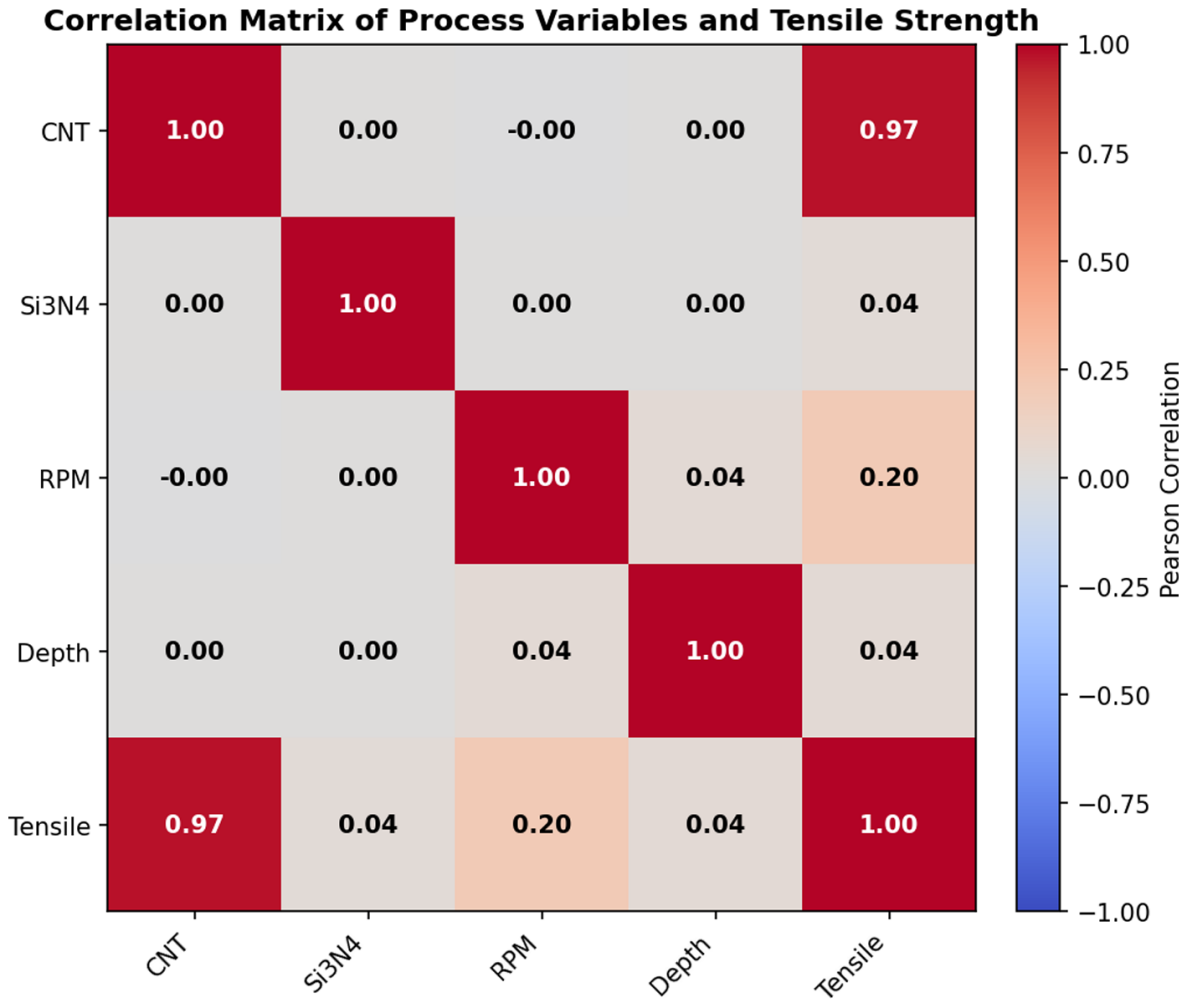
Pearson correlation matrix of the process variables and tensile strength.

### 2.5 Experimental procedure

The friction welding of the AA6061 tube plate and AA6063 tube was executed via a custom-designed vertical milling machine fitted with a tool holder, spindle, and backing block fixture. The apparatus permitted the HSS tool to traverse solely in the axial orientation while functioning at rotational velocities between 700 and 2000 rpm. The AA6061 plate was first processed into specimens of 50 mm × 50 mm × 6.3 mm, with a central aperture drilled for the AA6063 tube. Circumferential apertures were later created around the tube opening to enable the external incorporation of the CNT and Si₃N₄ reinforcements. The AA6063 tube was subsequently placed into the tube plate and firmly stabilized with a mild-steel backing block intended to avert displacement during the welding process. The specified amounts of CNT and Si₃N₄ powders, aligned with the chosen experimental parameters, were inserted into the circumferential apertures prior to welding. The HSS tool was turned at the specified velocity and progressively inserted into the workpiece to the required depth. The frictional heat produced by the revolving tool rendered the material pliable without liquefying it, allowing the material to migrate toward the weld contact and form a robust metallurgical bond between the tube and the tube plate. The welding procedure generally necessitated 30 to 60 s, with the plunge depth regulated by the machine spindle. Following the welding process, all samples were readied for tensile testing, and the tensile strength values obtained experimentally formed the dataset utilized for later machine learning evaluation.

### 2.6 Taguchi orthogonal experimental dataset array (L25)

A Taguchi L25 orthogonal array was utilized (Senthil Kumaran et al., 2020) to methodically structure experimental trials for the FWTPET procedure. Four process parameters, specifically CNT content, Si₃N₄ content, tool rotating speed, and plunge depth, were identified as the input variables. Every parameter was examined at five distinct levels, culminating in a total of 25 experimental trials. The Taguchi methodology determines optimal parameter combinations while minimizing the number of required experimental trials. The ultimate experimental input parameters employed for the fabrication of the welded specimens are delineated in Table 4.

**Table 4:** Final experimental input parameters used for the FWTPET process.

Sample no.	CNT (wt.%)	Si₃N₄ (wt.%)	Tool rotational speed (rpm)	Plunge depth (mm)
1	0.01	0.07	1,000	0.5
2	0.01	0.10	1,200	1.0
3	0.01	0.13	1,400	1.5
4	0.01	0.16	1,600	2.0
5	0.01	0.19	1800	2.5
6	0.02	0.07	1,200	1.5
7	0.02	0.10	1,400	2.0
8	0.02	0.13	1,600	2.5
9	0.02	0.16	1800	0.5
10	0.02	0.19	1,000	1.0
11	0.03	0.07	1,400	2.5
12	0.03	0.10	1,600	0.5
13	0.03	0.13	1800	1.0
14	0.03	0.16	1,000	1.5
15	0.03	0.19	1,200	2.0
16	0.04	0.07	1,600	1.0
17	0.04	0.10	1800	1.5
18	0.04	0.13	1,000	2.0
19	0.04	0.16	1,200	2.5
20	0.04	0.19	1,400	0.5
21	0.05	0.07	1800	2.0
22	0.05	0.10	1,000	2.5
23	0.05	0.13	1,200	0.5
24	0.05	0.16	1,400	1.0
25	0.05	0.19	1,600	1.5

### 2.7 Physics inspired machine learning framework

The proposed feature engineering strategy incorporates physics-inspired descriptors derived from experimentally measured process parameters. These descriptors are based on physically meaningful relationships associated with frictional heat generation, material flow, reinforcement loading, particle dispersion, and thermo-mechanical interactions occurring during the FWTPET process. Although these engineered variables are not directly derived from governing physical equations, they embed domain-specific knowledge into the machine learning framework, thereby improving the physical interpretability of the predictive models.

The engineered descriptors proposed in this research were designed to be physics inspired surrogate variables representing dominant thermo-mechanical phenomena in the FWTPET process. The mathematical formulations of these descriptors are based on friction welding mechanisms reported from literature although the descriptors are not directly derived from first principles equations. For instance, the Heat Generation Index (RPM × Depth) is the product of tool rotational speed and plunge depth, respectively, which both directly influence the frictional heat generation and plastic deformation during welding. Specific Energy Index (RPM/Depth) is a relative indicator of the rotational intensity input per unit plunge depth and indicates the balance of rotational energy input and material engagement. The Composite Heat Index (RPM × Depth) × (CNT + Si₃N₄) is a combination of thermal input and reinforcement loading that can be used to approximate coupled effects of heat generation and reinforcement incorporation within the stirred region. The Composite Ratio (CNT/Si₃N₄) likewise describes the relative contribution of carbon nanotubes and ceramic particles within the hybrid strengthening system, which affects the load transfer, dispersion of particles and strengthening behavior. These descriptors are thus useful in terms of their physical meaning for the dominant process mechanisms and in making the machine learning models more interpretable.

The material specifications, and experimental setup presented, including figures 1–4, should be referred from (Siddhardh et al., 2020). The experimental investigations presented in this research form the basis for developing and validating the proposed physics inspired machine learning framework for tensile strength prediction. Representative figures illustrating the experimental setup and procedure have been adapted (Siddhardh et al., 2020). The machine learning analyses, physics-inspired feature engineering, feature selection, model development, hyperparameter optimization, validation, XAI, and interpretation presented in this work are novel contributions of the current research.

## 3 Results and discussion

### 3.1 Dataset exploration and correlation analysis

Prior to developing machine learning models, there was an exploratory analysis of the experimental dataset to gain insights into how the process variables correlate with each other, and with tensile strength as well as their statistical properties and distribution. The dataset has 25 samples with four characteristics: CNT concentration, Si₃N₄ concentration, tool speed (RPM), and plunging depth with tensile strength as a final variable. Exploratory Data Analysis (EDA) was done to check how data is distributed, search for possible outliers, analyze features in relation to a target, estimate intervariable correlation, and establish some initial findings about the data to set the scene for later physics-oriented feature extraction and machine learning.

The histogram distributions of the four input variables CNT percentage, Si₃N₄ percentage, tool speed, and plunge depth and tensile strength results are shown in Figure 1. The experimental parameters have clear and balanced distributions in line with the Taguchi L25 experimental design, which means that all parameter levels are equally represented. The tensile strength data show a wide range of values and no extreme case of skewness, which confirms sufficient variability to allow for creating regression models.

Figure 2 presents the boxplots of the input parameters and tensile strength. According to the boxplots presented, there are no significant outliers in the dataset, which demonstrates that the data used in the research is sufficiently reliable. The relatively uniform distribution of input variables indicates that there is no outlier sample removal necessary for performing machine learning calculations.

The relationships between each process parameter and tensile strength are illustrated in the scatter plots where linear trend lines have been fitted in Figure 3. The variable that shows the highest positive relationship to tensile strength among the studied ones is CNT content. This indicates that increasing the amount of CNT can remarkably increase joint strength. In contrast, Si₃N₄ content, tool rotating speed, and plunge depth have relatively lower linear relationships, which means that their effects are affected by nonlinear interactions with the rest of the process parameters.

The Pearson correlation analysis for the experimental data is provided in Figure 4. The analysis shows that CNT content and tensile strength are strongly correlated (*r* = 0.97), suggesting that CNT is the most significant factor affecting joint strength. The correlation of tool rotating speed is weak (*r* = 0.20), while Si₃N₄ content (*r* = 0.04) and plunge depth (*r* = 0.04) are hardly correlated to the target variable at all. Moreover, a low correlation is found between input variables, which proves that the variables are not correlated and indicates their appropriateness for further feature engineering and machine learning process.

### 3.2 Physics inspired feature analysis

The original experimental variables, specifically CNT content, Si₃N₄ content, rotational speed (RPM) of the tool, and plunge depth, have information regarding the FWTPET process. However, these process parameters alone do not account for various processes which involve the component of thermal, mechanical, and material interactions during joint formation and development of tensile strength. Hence, a collection of physics-based engineered features has been designed according to previously known principles of friction welding and materials engineering. These engineered variables indicate physical phenomena such as production of friction heat, loading of reinforcing components, flow of material, distribution of reinforcement components, interaction of hybrid reinforcement materials, plastic deformation, and thermo-mechanical coupling. Through the introduction of engineered parameters, the machine learning models can learn not only from experimental data but also from the operating principles involved in the process.

Table 5 shows the first series of engineering features that come from experimental parameters. These features indicate the heat that the welded material receives and the amount of reinforcement brought into the welded area (the ratio of CNT to Si₃N₄). The Heat Generation Index measures the heat produced by both the rotation speed and the plunge depth. The Reinforcement Loading and Composite Ratio define the extent and proportion of composite reinforcements used in the welding process.

**Table 5:** Physics inspired features based on heat generation and reinforcement characteristics.

CNT	Si₃N₄	RPM	Depth	Heat generation index	Reinforcement loading	Composite ratio
0.01	0.07	1,000	0.5	500	0.08	0.142857
0.01	0.10	1,200	1.0	1,200	0.11	0.100000
0.01	0.13	1,400	1.5	2,100	0.14	0.076923
0.01	0.16	1,600	2.0	3,200	0.17	0.062500
0.01	0.19	1800	2.5	4,500	0.20	0.052632
0.02	0.07	1,200	1.5	1800	0.09	0.285714
0.02	0.10	1,400	2.0	2,800	0.12	0.200000
0.02	0.13	1,600	2.5	4,000	0.15	0.153846
0.02	0.16	1800	0.5	900	0.18	0.125000
0.02	0.19	1,000	1.0	1,000	0.21	0.105263
0.03	0.07	1,400	2.5	3,500	0.10	0.428571
0.03	0.10	1,600	0.5	800	0.13	0.300000
0.03	0.13	1800	1.5	2,700	0.16	0.230769
0.03	0.16	1,000	1.5	1,500	0.19	0.187500
0.03	0.19	1,200	2.0	2,400	0.22	0.157895
0.04	0.07	1,600	1.0	1,600	0.11	0.571429
0.04	0.10	1800	1.5	2,700	0.14	0.400000
0.04	0.13	1,000	2.0	2000	0.17	0.307692
0.04	0.16	1,200	2.5	3,000	0.20	0.250000
0.04	0.19	1,400	0.5	700	0.23	0.210526
0.05	0.07	1800	2.0	3,600	0.12	0.714286
0.05	0.10	1,000	2.5	2,500	0.15	0.500000
0.05	0.13	1,200	0.5	600	0.18	0.384615
0.05	0.16	1,400	1.0	1,400	0.21	0.312500
0.05	0.19	1,600	1.5	2,400	0.24	0.263158

The information that focuses on material flow characteristics in the FWTPET process is provided in Table 6. The Mixing Index describes how the fluid behavior of the material is influenced by the rotational speed and total amount of reinforcement. Composite Interaction demonstrates the level of interaction between CNT and Si₃N₄ particles. The Dispersion Index and Ceramic Dispersion Index monitor the performance of the distribution of CNT and Si₃N₄ particles at different rotational speeds. All these parameters will provide an estimate of the reinforcement dispersion mechanisms that are responsible for the reinforcement of the welded joint.

**Table 6:** Physics inspired features representing material flow and reinforcement dispersion.

CNT	Si₃N₄	RPM	Mixing index	Composite interaction	Dispersion index	Ceramic dispersion index
0.01	0.07	1,000	80	0.0007	10	70
0.01	0.10	1,200	132	0.0010	12	120
0.01	0.13	1,400	196	0.0013	14	182
0.01	0.16	1,600	272	0.0016	16	256
0.01	0.19	1800	360	0.0019	18	342
0.02	0.07	1,200	108	0.0014	24	84
0.02	0.10	1,400	168	0.0020	28	140
0.02	0.13	1,600	240	0.0026	32	208
0.02	0.16	1800	324	0.0032	36	288
0.02	0.19	1,000	210	0.0038	20	190
0.03	0.07	1,400	140	0.0021	42	98
0.03	0.10	1,600	208	0.0030	48	160
0.03	0.13	1800	288	0.0039	54	234
0.03	0.16	1,000	190	0.0048	30	160
0.03	0.19	1,200	264	0.0057	36	228
0.04	0.07	1,600	176	0.0028	64	112
0.04	0.10	1800	252	0.0040	72	180
0.04	0.13	1,000	170	0.0052	40	130
0.04	0.16	1,200	240	0.0064	48	192
0.04	0.19	1,400	322	0.0076	56	266
0.05	0.07	1800	216	0.0035	90	126
0.05	0.10	1,000	150	0.0050	50	100
0.05	0.13	1,200	216	0.0065	60	156
0.05	0.16	1,400	294	0.0080	70	224
0.05	0.19	1,600	384	0.0095	80	304

Table 7 provides an overview of engineered features that contribute to energy concentration and reinforcement density during the welding process. Specific Energy Index shows the amount of rotational power used for each plunge depth and is a sign of localized plastic deformation. The Composite Heat Index combines frictional heating and the amount of reinforcement used to assess the influence of both thermal energy and reinforcement. Reinforcement Density shows how concentrated the reinforcement particles are in the deformation area and indicates how effective the reinforcement distribution is.

**Table 7:** Physics inspired features associated with energy distribution and thermo-mechanical coupling.

RPM	Depth	CNT	Si₃N₄	Specific energy index	Composite heat index	Reinforcement density
1,000	0.5	0.01	0.07	2000.000	40	0.160
1,200	1.0	0.01	0.10	1200.000	132	0.110
1,400	1.5	0.01	0.13	933.333	294	0.093
1,600	2.0	0.01	0.16	800.000	544	0.085
1800	2.5	0.01	0.19	720.000	900	0.080
1,200	1.5	0.02	0.07	800.000	162	0.060
1,400	2.0	0.02	0.10	700.000	336	0.060
1,600	2.5	0.02	0.13	640.000	600	0.060
1800	0.5	0.02	0.16	3600.000	162	0.360
1,000	1.0	0.02	0.19	1000.000	210	0.210
1,400	2.5	0.03	0.07	560.000	350	0.040
1,600	0.5	0.03	0.10	3200.000	104	0.260
1800	1.5	0.03	0.13	1200.000	432	0.107
1,000	1.5	0.03	0.16	666.667	285	0.127
1,200	2.0	0.03	0.19	600.000	528	0.110
1,600	1.0	0.04	0.07	1600.000	176	0.110
1800	1.5	0.04	0.10	1200.000	378	0.093
1,000	2.0	0.04	0.13	500.000	340	0.085
1,200	2.5	0.04	0.16	480.000	600	0.080
1,400	0.5	0.04	0.19	2800.000	161	0.460
1800	2.0	0.05	0.07	900.000	432	0.060
1,000	2.5	0.05	0.10	400.000	375	0.060
1,200	0.5	0.05	0.13	2400.000	108	0.360
1,400	1.0	0.05	0.16	1400.000	294	0.210
1,600	1.5	0.05	0.19	1066.667	576	0.160

In this research, all the physics-based features designed have been summarized in Table 8 along with their mathematical expressions as well as interpretations in the physical sense. Each of the developed features represents one of the concepts relating to events taking place during the process of FWTPET, such as heat generation, the flow of the substance, interaction of reinforcements, dispersion of reinforcements, thermo-mechanical coupling, and plastic deformation. It should be noted that this set of features allows the feature space to be interpreted in physical terms thus facilitating future feature validation, model design, and XAI-related activities.

**Table 8:** Summary of physics inspired engineered features and their physical significance.

Feature name	Formula	Physical interpretation
Heat Generation Index	RPM × Depth	Proxy for frictional heat input due to combined rotational speed and plunge depth.
Reinforcement Loading	CNT + Si₃N₄	The total reinforcement fraction contributes to load transfer and strengthening.
Composite Ratio	CNT / Si₃N₄	Relative contribution of CNT with respect to ceramic reinforcement.
Mixing Index	RPM × (CNT + Si₃N₄)	Indicator of stirring intensity and reinforcement dispersion.
Composite Interaction	CNT × Si₃N₄	Captures the synergistic interaction between CNT and Si₃N₄ reinforcements.
Dispersion Index	RPM × CNT	Represents rotational influence on CNT distribution.
Ceramic Dispersion Index	RPM × Si₃N₄	This represents the rotational influence on the dispersion of Si₃N₄ particles.
Specific Energy Index	RPM / Depth	Rotation intensity per unit plunge depth, indicating localized plastic deformation.
Composite Heat Index	(RPM × Depth) × (CNT + Si₃N₄)	This is a combined descriptor for both thermal energy and reinforcement loading.
Reinforcement Density	(CNT + Si₃N₄) / Depth	Approximate reinforcement concentration within the deformation zone.

The Pearson correlation matrix of the original experimental variables and the engineered variables proposed in this work is shown in Figure 5. This analysis gives a detailed overview of the linear relationships between the process parameters, engineered descriptors and the target variable (tensile strength). The engineered feature space contains better and more meaningful relationships between its features and tensile strength compared to the original feature space, due to the inclusion of thermal, material flow, reinforcement interaction, and thermo-mechanical coupling phenomena.

**Figure 5:**
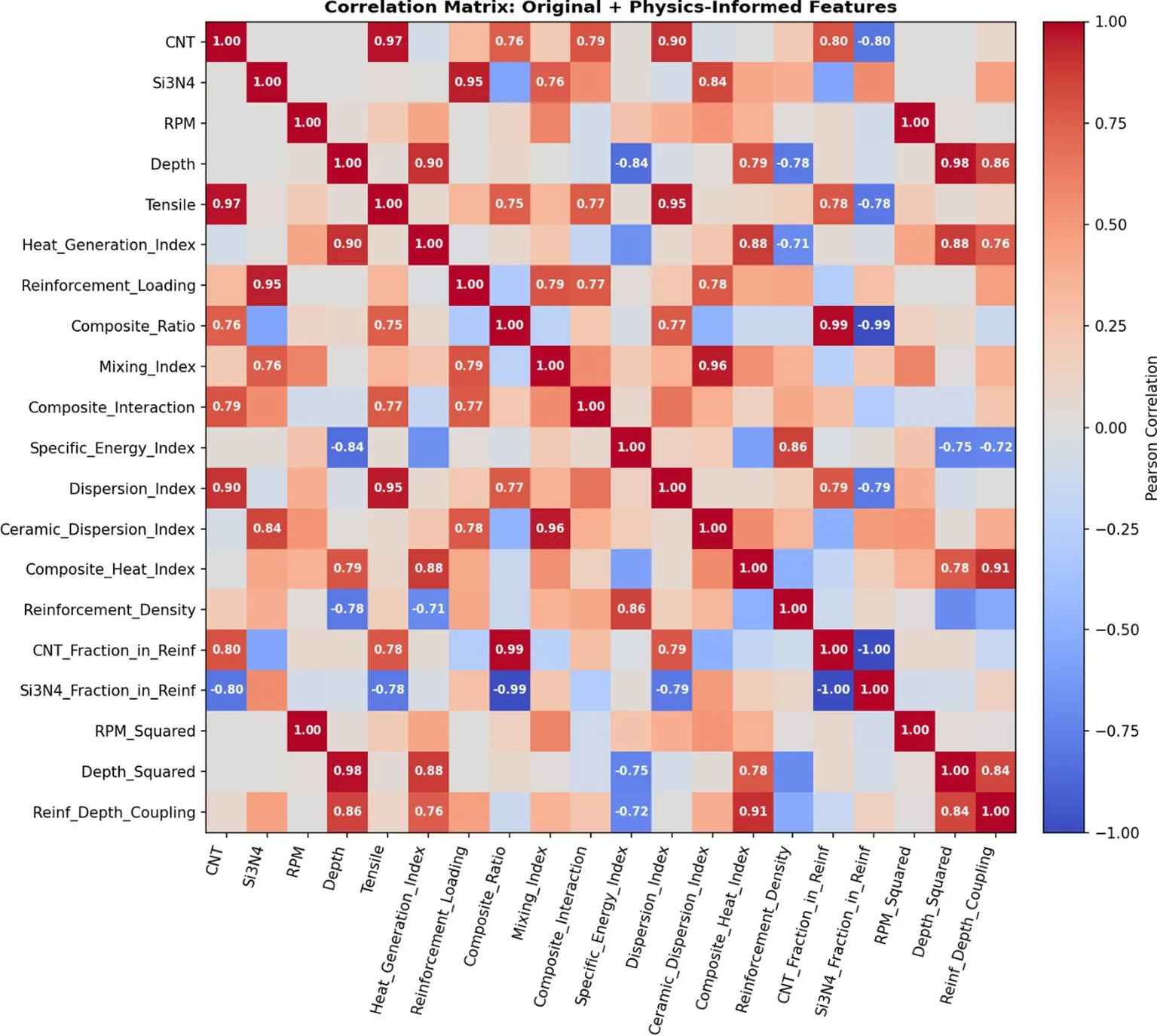
Correlation matrix of original and physics inspired engineered features.

The correlation matrix shows that the tensile strength has the highest positive correlation with CNT content (about *r* = 0.97), which means that the main contribution of the CNTs is to improve the mechanical properties of the welded joint. Some of the designed variables also show favorable positive correlations with tensile strength. The relationships of the following indices with the target variable are significant: Dispersion Index (≈0.95), Heat Generation Index (≈0.90), Composite Heat Index (≈0.79), Mixing Index (≈0.77), Composite Interaction (≈0.77), and Composite Ratio (≈0.75). Based on these results, it is concluded that the contribution of frictional heat and reinforcement dispersion, hybrid reinforcement interaction and thermo-mechanical coupling are significant factors affecting the tensile strength.

There are also several expected relationships among the engineered descriptors in the correlation matrix. For example, the Heat Generation Index is highly correlated with the Composite Heat Index, and the Mixing Index is correlated with the Ceramic Dispersion Index, indicating that the dispersion of reinforcement depends on the rotational speed. Likewise, the Composite Ratio and reinforcement fraction descriptors have good positive and negative correlation values due to its complementary reinforcement proportion descriptors. Relationships indicate that the designed features can capture physically meaningful process features rather than arbitrary mathematical transformations.

The results of the correlation analysis indicate that the proposed physics inspired feature engineering procedure is relevant in enriching the original experimental data with feature descriptors that are more representative of the welding physics. These features offer a more robust and explainable basis for further feature selection, machine learning model training and XAI analysis.

Through the physics inspired feature analysis, it is shown that redefining the original experimental variables to physically meaningful features significantly improve the representation of FWTPET process. The basic input parameters, namely, CNT content, Si₃N₄ content, tool rotational speed and plunge depth, are the basic experimental information, however, they do not explicitly characterize the complex thermo-mechanical phenomena occurring during friction welding. The features thus developed are therefore intended to depict significant physical mechanisms such as frictional heat generation, material flow, reinforcement loading, reinforcement dispersion, interaction between hybrid reinforcement, localized plastic deformation and thermo-mechanical coupling.

The correlation analysis also shows that several engineered features have higher correlations with tensile strength than the original process features. The Heat Generation Index, Dispersion Index, Mixing Index, Composite Heat Index, and Composite Interaction are all features that successfully address the simultaneous effect of the three rotational, plunge, and reinforcement variables on the formation of the joints. They provide good correlation with the target variable and the tensile strength of the welded joints appears to be controlled by two or more coupled physical mechanisms, not by any single process parameter.

The engineered descriptors are also physically interpretable. The Heat Generation Index accounts for frictional heat generated by softening of the material, the Mixing Index indicates the transport of the reinforcements in the stirring process, the Composite Interaction indicates the synergistic effect of two reinforcements, CNT and Si₃N₄, the Dispersion Indices estimates the distribution of both reinforcements in the plasticized region, and the Composite Heat Index indicates the simultaneous effect of thermal energy and reinforcements loading. As such, there is a direct scientific interpretation for each engineered feature, related to the FWTPET process.

In addition, the correlation matrix shows little correlation between the original experimental variables and important relationships between the engineered ones. Such relationships show that this proposed feature-engineering framework is effective in incorporating domain knowledge into the dataset, not by using arbitrary feature construction. This creates a richer feature space with additional information about the processes, which can lead to greater accuracy and better model interpretation.

The proposed physics inspired feature engineering framework ultimately connects the experimental process parameters to the physics of the material. The features are derived from known principles of friction welding, allowing the regression models to learn more meaningful relationships in the machine learning workflow and facilitating the reliable analysis of XAI for tensile strength prediction.

### 3.3 Feature selection and validation

After developing physics inspired engineered features, a comprehensive feature validation and selection procedure was applied to pinpoint the most substantial and informative descriptors dictating the tensile strength predictions. The proposed feature engineering framework utilized several derived variables rooted in physical principles, so it was necessary to establish the statistical significance and predictive power of each newly engineered feature.

Validation of the engineered features was conducted using various complementary techniques such as Pearson correlation, Spearman rank correlation, Mutual Information (MI), Variance Inflation Factor (VIF) analysis, Boruta feature selection, and SHAP analysis. Pearson correlation is suitable for measuring linear dependencies between each feature and tensile strength, while Spearman correlation quantifies monotonic relationships that are not always linear. Mutual Information was used to analyze non-linear interactions between engineered descriptors and the variable of interest. VIF was calculated to discover redundant features with collinearity. Finally, Boruta feature selection and SHAP analysis were used to rank original and engineered features in terms of their predictive power and keep only those descriptors that are physically meaningful and statistically relevant in the model.

Figure 6 shows both the Pearson and Spearman correlation heatmap for the original process parameters and the suggested physics inspired engineered features. The Pearson heatmap depicts the strength of linear correlations between variables while the Spearman heatmap describes monotonic correlations without requiring the linearity count. The CNT content and the Dispersion Index have the highest correlation with tensile strength from both analyses. Also, the generated features, including Composite Interaction, Composite Ratio, and Mixing Index, show the considerable relationship with the target.

**Figure 6:**
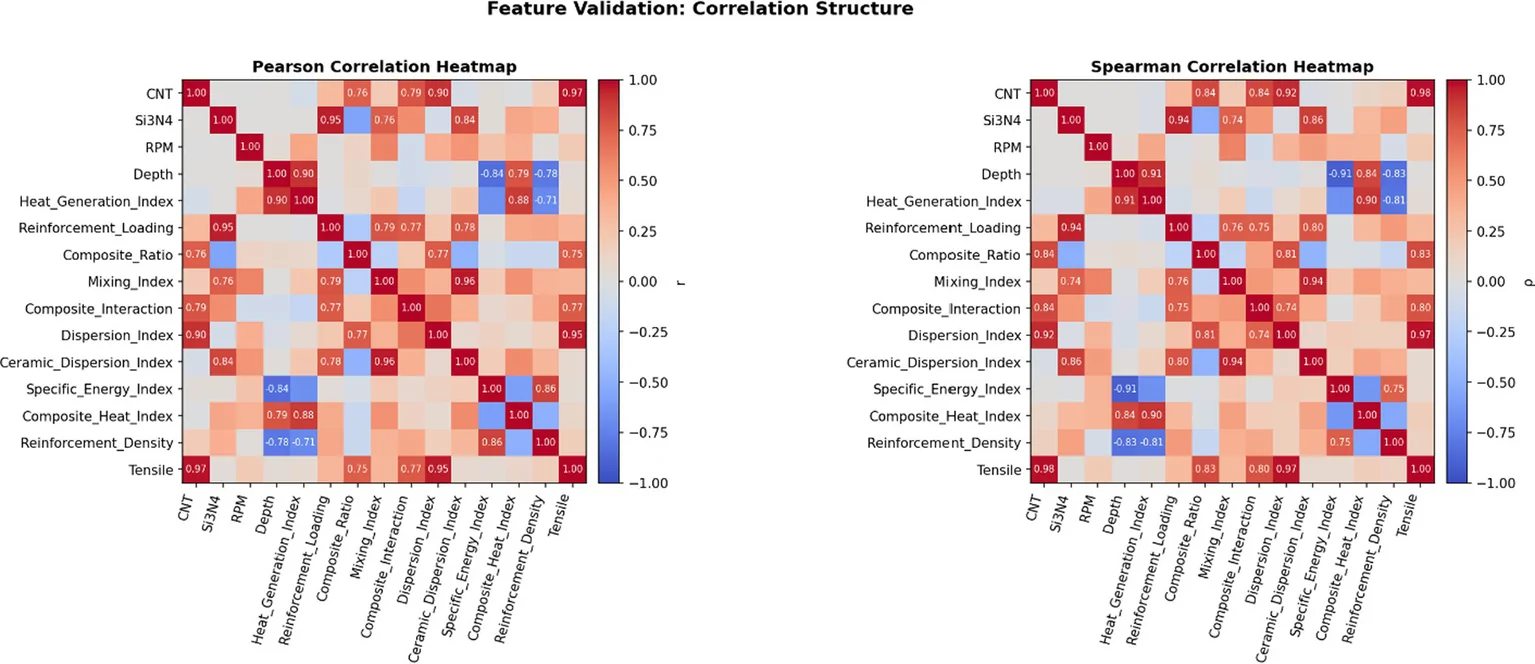
Pearson and Spearman correlation heatmaps for feature validation.

Table 9 shows summary of the ranking of every original as well as modified features procured through Pearson correlation, Spearman correlation, Mutual Information and general average ranking. CNT concentration has the highest rating among all features, while other distinguished features include Dispersion Index, Composite Interaction and Composite Ratio. The results show that the features which are correlated to the distribution of reinforcement and interaction of hybrids provide the greatest contributions to the prediction of tensile strength. Meanwhile, those features that correspond to rotational speed, plunge depth, or single Si₃N₄ concentration have lower rank among other established features.

**Table 9:** Feature ranking based on pearson correlation, spearman correlation, mutual information, and average rank.

Rank	Feature	Pearson correlation	Spearman correlation	Mutual information	Average rank
1	CNT	0.9707	0.9806	1.4796	1.0000
2	Dispersion Index	0.9548	0.9746	1.0445	2.0000
3	Composite Interaction	0.7668	0.8015	0.4661	3.3333
4	Composite Ratio	0.7518	0.8262	0.4529	3.6667
5	Reinforcement Loading	0.3415	0.3141	0.0000	7.1667
6	Mixing Index	0.3407	0.3231	0.0000	7.1667
7	Reinforcement Density	0.1719	0.1401	0.0160	7.6667
8	RPM	0.1994	0.1961	0.0000	8.1667
9	Specific Energy Index	0.0499	0.0719	0.0622	9.0000
10	Composite Heat Index	0.1017	0.1724	0.0000	9.1667
11	Ceramic Dispersion Index	0.0696	0.0558	0.0000	10.5000
12	Heat Generation Index	0.0572	0.0458	0.0000	11.1667
13	Depth	0.0446	0.0079	0.0000	12.1667
14	Si₃N₄	0.0365	0.0000	0.0000	12.8333

To quantify the multicollinearity between the original and engineered features, VIF analysis was carried out and presented in Table 10. The high VIF values are to be expected, as some of the engineered descriptors are mathematical combinations of the experimental variables. While there are clear interdependencies between these features, each one is a unique physical phenomenon, including frictional heat generation, reinforcement loading, reinforcement dispersion, or thermo-mechanical coupling. Thus, the observed multicollinearity is not considered as redundant information but is rather seen as the physical relationships inherent in the feature engineering framework proposed.

**Table 10:** Variance inflation factor (VIF) analysis of original and engineered features.

Feature	VIF	Multicollinearity level
CNT	∞	High
Si₃N₄	∞	High
Reinforcement Loading	∞	High
Ceramic Dispersion Index	∞	High
Dispersion Index	∞	High
Mixing Index	∞	High
Heat Generation Index	356.921	High
Composite Interaction	167.570	High
RPM	146.356	High
Depth	130.921	High
Composite Heat Index	113.351	High
Reinforcement Density	90.167	High
Specific Energy Index	66.275	High
Composite Ratio	61.182	High

The results from the feature validation experiments clearly show that the proposed physics inspired engineered features are significantly more useful for representing the FWTPET process than are the original experimental features from the experiments themselves. When evaluating all descriptors, the content of CNT proved to be the most significant one with the highest Pearson correlation (0.9707), Spearman correlation (0.9806) and Mutual Information score (1.4796) for the tensile strength. This observation shows that the load transfer, grain refinement and crack resistance improvement in the welded joint is largely due to the presence of CNT reinforcement. The Dispersion Index was also in second place among the feature selection approaches, showing the significance of the distribution of reinforcements in the friction welding process.

This feature is a combination of both CNT content and rotational speed, which is a positive indicator of the capacity of the rotating tool to uniformly disperse nanotubes in the plasticized material. Better dispersion reduces the tendency for particle agglomeration and results in more uniform strengthening, which enhances tensile strength. Additionally, the Composite Interaction and Composite Ratio showed good predictive ability. These descriptors reflect the synergistic interaction between CNT and Si₃N₄ reinforcements, which is not the case if they are treated separately. The high ranks suggest that the reinforcement contents contribute more to the joint strength when combined with the hybrid reinforcements than when considered individually. Interestingly, the individual correlations of the original process parameters with tensile strength were relatively low: RPM, plunge depth and content of Si₃N₄. This indicates that their effect is more indirect and is more obvious when expressed as a physically relevant combination (e.g., the Heat Generation Index, Mixing Index and Composite Heat Index).

Thus, the engineered descriptors are more complete representations of the underlying thermo-mechanical phenomena that take place during the FWTPET process. The VIF analysis reveals significant correlations between some engineered features, indicating the presence of multicollinearity. This is normal because the suggested descriptors are based on physical variables commonly used in labs to observe various aspects of the same phenomena. In contrast to classical feature engineering where high multicollinearity is usually considered as a sign of redundancy, the current framework keeps these descriptors, since each one has specific physical meaning. The Heat Generation Index is a measure of the frictional heat input, while the Mixing Index is a measure for reinforcement transport, both of which are dependent on the rotational speed.

Likewise, the Composite Heat Index combines the effects of thermal load with reinforcement loading that is a unique thermo mechanical characteristic. The feature validation process validates the physics inspired descriptors, which successfully connect the experimental process parameters and the physics underlying the tensile strength, on average. Pearson correlation, Spearman correlation and Mutual Information consistently rank the same features, showing the strength of the engineered features, and the VIF analysis shows that these features are physically coupled. The feature space generated by these validated descriptors is interpretable and scientifically meaningful, laying the groundwork for the next step of machine learning model development and SHAP based explainability analysis.

The results from the VIF analysis indicated considerable multicollinearity among some engineered descriptors, which can be explained by the fact that these variables have mostly been mathematically calculated from the same experimental process parameters. Therefore, VIF results were examined as a means of diagnosing feature overlaps rather than a basis for eliminating variables from the analysis. Since the final predictive model relies mainly on tree-based machine learning methods that are characteristically less sensitive to multicollinearity, it is anticipated that the correlated features will not pose any significant hindrance to the model’s performance, even though this limitation has been recognized and should be taken into account in interpreting the relationships between the variables.

The entire feature ranking resulting from the interaction between Recursive Feature Elimination, Boruta feature selection, permutation importance, and SHAP feature importance is documented in Table 11. Consensus scoring ranks the original parameters and physics inspired engineered features by their relevancy to prediction of tensile strength. The ranking shows that CNT content is the most important descriptor, followed by Dispersion Index, Composite Ratio, and Composite Interaction, confirming that descriptors related to reinforcement processes strongly influence the tensile strength predictions. The agreement between several selection methods verifies the validity and stability of physics inspired feature engineering.

**Table 11:** Unified feature ranking obtained using multiple feature selection techniques.

Feature	Feature group	RFE rank	Boruta rank	Boruta confirmed	Boruta tentative	Permutation mean	Permutation STD	Mean absolute SHAP	Consensus rank
CNT	Original	1	1	Yes	No	0.7536	0.1824	26.6542	1.375
Dispersion Index	Physics	2	1	Yes	No	0.1857	0.0419	10.9584	2.125
Composite Ratio	Physics	3	1	Yes	No	0.0110	0.0030	2.5703	3.125
Composite Interaction	Physics	4	1	Yes	No	0.0148	0.0040	2.5145	3.375
Composite Heat Index	Physics	5	2	No	No	0.0020	0.0009	0.4388	6.625
RPM	Original	8	5	No	No	0.0031	0.0015	0.7996	6.875
Reinforcement Loading	Physics	7	3	No	No	0.0013	0.0005	0.2246	8.125
Specific Energy Index	Physics	6	7	No	No	0.0006	0.0004	0.2206	9.125
Mixing Index	Physics	11	7	No	No	0.0008	0.0003	0.2793	9.375
Reinforcement Density	Physics	9	2	No	No	0.0005	0.0004	0.3626	9.625
Heat Generation Index	Physics	12	9	No	No	0.0008	0.0003	0.1871	10.375
Ceramic Dispersion Index	Physics	10	5	No	No	0.0006	0.0003	0.2089	10.625
Si₃N₄	Original	13	8	No	No	0.0008	0.0003	0.1613	11.375
Depth	Original	14	10	No	No	0.0003	0.0003	0.1082	12.875

The comparison between the original experimental variables and physics inspired engineered features is shown in Table 12. The comparison illustrates that engineered features group scored lower mean in ranking and produced a larger number of descriptors with the highest ranks than the original feature set. In other words, physics-driven features are more effective in discovering the underlying thermo-mechanical phenomena related to tensile strength than the original values.

**Table 12:** Comparison between original and physics inspired feature groups.

Feature group	Number of features	Mean consensus rank	Best consensus rank	Number of features in top 5
Original	4	8.125	1.375	1
Physics-Informed	10	7.250	2.125	3

Information in Table 13 provides summarized data regarding the most important features selected with the help of Recursive Feature Elimination approach. According to RFE, the method works by systematically eliminating variables that provide the least amount of information while retaining the ones that help maximize prediction efficiency. The results obtained indicate that CNT, Dispersion Index, Composite Ratio, and Composite Interaction are important parameters in tensile strength prediction.

**Table 13:** Top features identified by recursive feature elimination (RFE).

Rank	Feature	RFE rank	RFE score
1	CNT	1	1.0000
2	Dispersion Index	2	0.9231
3	Composite Ratio	3	0.8462
4	Composite Interaction	4	0.7692
5	Composite Heat Index	5	0.6923
6	Specific Energy Index	6	0.6154
7	Reinforcement Loading	7	0.5385

Table 14 summarizes the feature importance obtained through permutation analysis. Permutation analysis measures the drop in prediction ability due to disrupting the values of the features. According to the results, it can be implied that CNT proves to be the most important factor in the performance of the model, and Dispersion Index follows with less influence, while other descriptors are less effective in this respect.

**Table 14:** Top features identified by permutation importance.

Rank	Feature	Permutation mean	Permutation STD	Permutation score
1	CNT	0.7536	0.1824	1.0000
2	Dispersion Index	0.1857	0.0419	0.2461
3	Composite Interaction	0.0148	0.0040	0.0192
4	Composite Ratio	0.0110	0.0030	0.0142
5	RPM	0.0031	0.0015	0.0037
6	Composite Heat Index	0.0020	0.0009	0.0023
7	Reinforcement Loading	0.0013	0.0005	0.0013

Table 15 illustrates the global feature importance based on SHapley Additive exPlanations (SHAP) method. Using this method, the average value of SHAP can be calculated and applied to cost-effective predictions without losing the meaning of the parameters. The results obtained confirm that CNT plays the key role in the alterations of the tensile strength.

**Table 15:** Top features identified using SHAP analysis.

Rank	Feature	Mean absolute SHAP	SHAP score
1	CNT	26.6542	1.0000
2	Dispersion Index	10.9584	0.4087
3	Composite Ratio	2.5703	0.0927
4	Composite Interaction	2.5145	0.0906
5	RPM	0.7996	0.0260
6	Composite Heat Index	0.4388	0.0125
7	Reinforcement Density	0.3626	0.0096

The results of the Boruta feature selection algorithm are summarized in Table 16, showing the variables that have some statistical significance, since they are more important than the randomized shadow variables. The algorithm revealed that only the descriptors CNT, Dispersion Index, Composite Ratio and Composite Interaction were strongly relevant to the data, while the rest showed relatively low statistical significance. The Boruta results also corroborate the proposed effective physics inspired feature engineering strategy. The results of the feature selection and validation analysis showcase the ability of the proposed physics inspired feature engineering framework to select the most relevant features that have the greatest impact on tensile strength prediction.

**Table 16:** Top features selected using Boruta feature selection.

Rank	Feature	Boruta rank	Confirmed	Tentative	Boruta score
1	CNT	1	Yes	No	1.0
2	Composite Interaction	1	Yes	No	1.0
3	Composite Ratio	1	Yes	No	1.0
4	Dispersion Index	1	Yes	No	1.0
5	Composite Heat Index	2	No	No	0.2
6	Reinforcement Density	2	No	No	0.2
7	Reinforcement Loading	3	No	No	0.2

To thoroughly assess the relevance and robustness of the features, multiple feature selection approaches were applied, such as Pearson correlation, Spearman correlation, Mutual Information, Recursive Feature Elimination (RFE), Boruta, Permutation Importance, SHAP analysis, and VIF analysis. The results indicates that the content of the CNT is the most influential feature selected by all the feature selection methods among the tensile strength. Furthermore, the features that were engineered (Dispersion Index, Composite Ratio, Composite Interaction) were always among the top performing features, indicating that the dispersion of reinforcement and interaction with reinforcement is more influential than the process parameters themselves. These descriptors are representative of important physical phenomena such as reinforcement distribution in the material, flow of material and synergistic strengthening mechanisms during FWTPET process.

Original and engineered feature groups are compared to further support that the proposed physics inspired descriptors are superior. While just four parameters of the original process were used, most of the top-ranked variables were from the engineered feature group, demonstrating the value of incorporating domain-specific physical knowledge into the machine learning process. This finding shows that the physics inspired feature engineering significantly complements the experimental data and offers more meaningful descriptions for predictive modeling. VIF analysis indicated that there was a significant multicollinearity between certain engineered features. This is, however, a natural phenomenon, as many of the descriptors are derived from common experimental parameters.

These correlated features describe the physical aspects of the welding process, not redundant information, but frictional heat generation, thermo-mechanical coupling, reinforcement loading and plastic deformation. Hence, these engineered descriptors were kept because they have different physical meanings and provide different information to the predictive model. In general, the Pearson correlation, Spearman correlation, Mutual Information, Boruta, RFE, Permutation Importance and SHAP analysis results indicates a high level of agreement, which indicates the robustness and reliability of the feature engineering strategy. The feature set validated not only helps to increase the predictability of the model but also contributes to the model interpretability by corroborating the statistical importance with physical mechanisms of friction welding. As a result, the chosen physics inspired descriptors lay a scientifically sound basis for the following machine learning model development, hyperparameter optimization and XAI analysis.

Consensus ranking, based on RFE, Boruta feature selection, Permutation Importance and SHAP analysis, is used to find the top 10 features with the highest influence, which are shown in Figure 7. The horizontal bar chart shows the features ranked by the consensus rank with a smaller number being more important for predicting tensile strength. The bars are color coded, with the original experimental variables colored blue and the physics informed engineered features colored red. As can be seen, the content of CNT is the feature that is most influential, with lowest consensus rank (1.375) among all variables.

**Figure 7:**
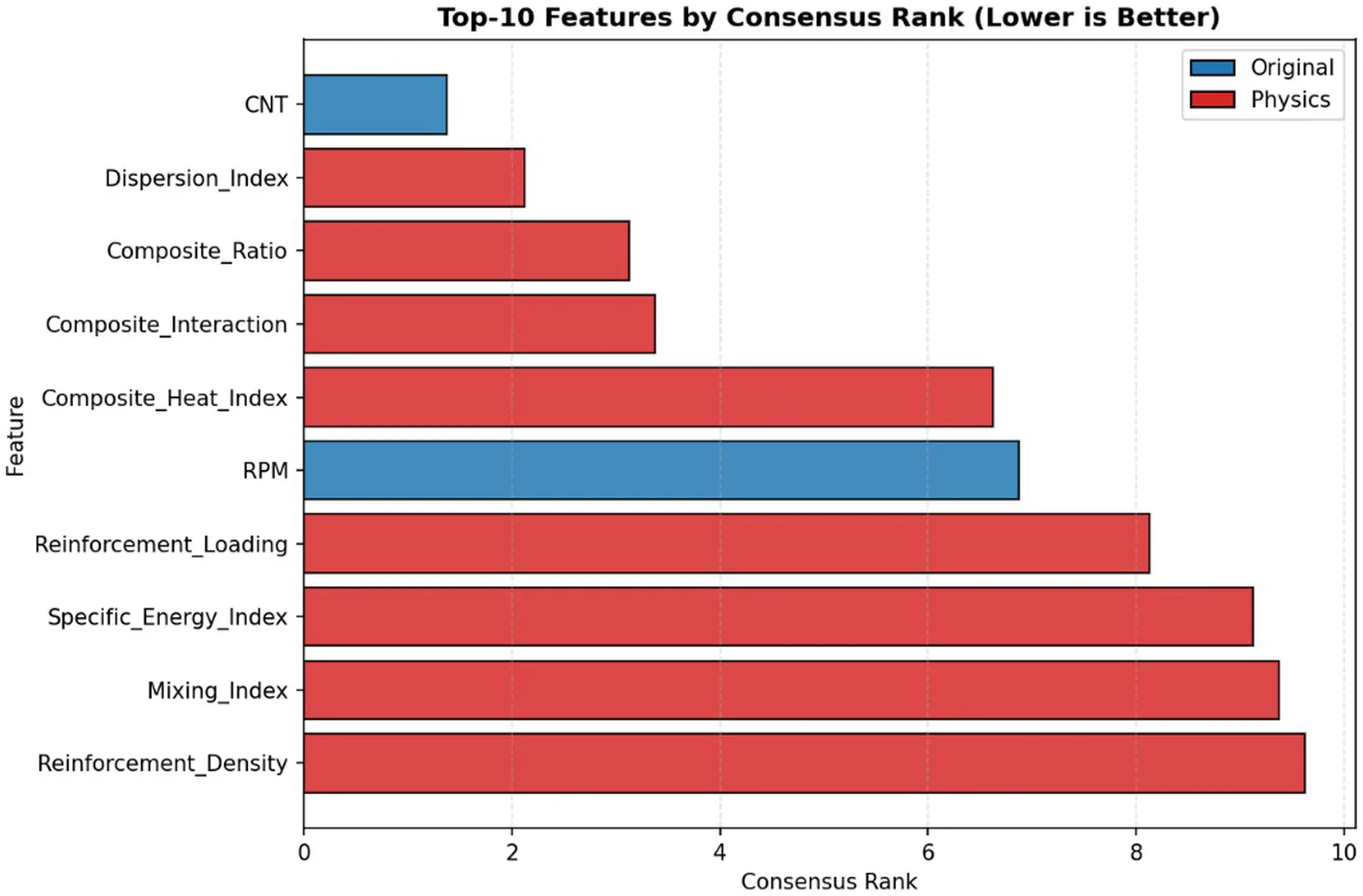
Top 10 features ranked by consensus feature selection methods.

This result indicates that the amount of carbon nanotube reinforcement has the greatest impact on the tensile strength of the FWTPET joints. The grain refinement provided by the CNT reinforcement, the load transfer between reinforcement and aluminum matrix and the crack resistance of CNT reinforcement are significant improvements that greatly increase joint strength. The Dispersion Index is the second engineered descriptor, which shows that the even distribution of the CNT’s because of the rotational speed of the tool has a significant impact on the mechanical properties of the weld joint. A satisfactory dispersion of reinforcement reduces particle agglomeration, and a more uniform microstructure provides better tensile properties. The third is the Composite Ratio and the fourth is the Composite Interaction.

They describe the relative contribution and interaction of reinforcement CNT and Si₃N₄. The high ranking shows that including both hybrid reinforcements has a greater effect on tensile strength than both reinforcements alone. The findings of this observation validate the hybrid reinforcement method used in the FWTPET process. The fifth ranked feature is the Composite Heat Index, which is a composite of the frictional heat generation and reinforcement loading. This describes the thermo-mechanical conditions associated with plastic deformation and the mixing of material during welding. It does not play as significant a role as the CNT-related features, but it does add significantly to the predictive performance. Remarkably, RPM, which was one of the original experimental variables, ranks only sixth overall.

This implies that tensile strength can not only be varied by rotation speed itself, but also by other factors. However, its effect is more significant when it is considered as part of reinforcement features which are given greater rank, such as the Dispersion Index and Composite Heat Index. The other three most important descriptors (Reinforcement Loading, Specific Energy Index, Mixing Index, and Reinforcement Density) also stem from the suggested physics inspired feature engineering framework. These variables together describe reinforcement concentration, energy distribution, stirring intensity and dispersion of reinforcement in the plasticized weld zone.

The presence of these descriptors among the top 10 features indicates that the descriptors derived from the physical process can be more informative and richer than the process parameters themselves. It is worth noting that 8 of the 10 most-ranked features are physics-driven engineered features, and only 2 features are the original experimental features (CNT and RPM). The results show the power of introducing domain specific physical knowledge into the feature engineering process. The engineered characteristics are effective at encoding the underlying mechanisms of frictional heat generation, material flow, reinforcement interaction, and plastic deformation which control the process of joint formation, without depending on the direct measure of process parameters.

Figure 7 verifies that the proposed physics inspired feature engineering approach leads to significantly improved and more predictive features. The results of multiple feature selection methods show engineered descriptors are superior to most original variables, giving a scientifically interpretable feature space for the machine learning modelling and XAI analysis. The results in this research confirm the central thesis of this work that the addition of physically meaningful descriptors produces more powerful, accurate and interpretable tensile strength prediction models.

Table 17 illustrates the unified feature ranking obtained because of the application of four complementary methodologies of feature selection, which are RFE, Boruta feature selection, Permutation Importance, and SHAP feature importance analysis. The consensus ranking provides the average significance in terms of predictive value of each feature across the methods, and thus it offers a reliable evaluation of predictive significance. It can be observed that CNT is still the most significant predictor among other descriptors, which are Dispersion Index, Composite Interaction, and Composite Index. Therefore, one can conclude that the presented physics inspired descriptors outperform almost all original variables from the processing.

**Table 17:** Unified consensus ranking of original and physics inspired features.

Feature	Feature group	RFE rank	Boruta rank	Boruta confirmed	Boruta tentative	Permutation mean	Permutation STD	Mean absolute SHAP	Consensus rank
CNT	Original	1	1	Yes	No	0.7711	0.1990	27.0075	1.375
Dispersion Index	Physics	2	1	Yes	No	0.1935	0.0499	10.9566	2.125
Composite Interaction	Physics	4	1	Yes	No	0.0154	0.0058	2.4558	3.125
Composite Ratio	Physics	3	1	Yes	No	0.0086	0.0032	2.3159	3.375
RPM	Original	6	6	No	No	0.0021	0.0009	0.4534	6.250
Reinforcement Density	Physics	5	3	No	No	0.0005	0.0004	0.2451	6.250
Composite Heat Index	Physics	7	4	No	No	0.0020	0.0007	0.3224	6.500
Specific Energy Index	Physics	9	5	No	No	0.0004	0.0003	0.1931	8.250
Mixing Index	Physics	11	2	No	No	0.0002	0.0002	0.1030	9.250
Reinforcement Loading	Physics	8	7	No	No	0.0004	0.0002	0.0948	9.500
Ceramic Dispersion Index	Physics	10	8	No	No	0.0002	0.0001	0.0640	10.750
Si₃N₄	Original	12	9	No	No	0.0002	0.0003	0.0954	11.250
Heat Generation Index	Physics	13	10	No	No	0.0001	0.0001	0.0366	13.500
Depth	Original	14	11	No	No	0.0002	0.0001	0.0552	13.500

In Table 18, the predictive potential of the original experimental variables compared with that of the new physics inspired engineered features. While there were only four original variables, 10 engineered features were created using known principles of FW technology. The results show that the physics inspired features had a lower average ranking and provided three out of five most prominent features, thus proving that physical variables are more complete and provide more information about the FWTPET process compared to initial experimental variables.

**Table 18:** Comparison between original and physics inspired feature groups.

Feature group	Number of features	Mean consensus rank	Best consensus rank	Features in top five
Original	4	8.0938	1.375	1
Physics-Informed	10	7.2625	2.125	3

In Table 19, the 10 best features selected using the combined feature-selection methods. The ranking was based on the findings of RFE, Boruta, Permutation Importance, and SHAP analysis. The data shows that features connected with the reinforcement properties, reinforcement distribution, and interaction of hybrid reinforcements were the most prominent in the prediction of the process. Eight out of 10 top features were the ones proposed using physics-based feature engineering methods.

**Table 19:** Top 10 features selected by the consensus feature selection framework.

Rank	Feature	Feature group	Consensus rank
1	CNT	Original	1.375
2	Dispersion Index	Physics-Informed	2.125
3	Composite Interaction	Physics-Informed	3.125
4	Composite Ratio	Physics-Informed	3.375
5	RPM	Original	6.250
6	Reinforcement Density	Physics-Informed	6.250
7	Composite Heat Index	Physics-Informed	6.500
8	Specific Energy Index	Physics-Informed	8.250
9	Mixing Index	Physics-Informed	9.250
10	Reinforcement Loading	Physics-Informed	9.500

The VIF analysis revealed substantial multicollinearity among several engineered descriptors because many of these variables were mathematically derived from the same experimental process parameters. Although the proposed tree-based machine learning models are relatively robust to multicollinearity in terms of predictive performance, correlated features may influence feature importance rankings and SHAP-based interpretations by distributing importance across related descriptors. The engineered features were intentionally retained to investigate different physically motivated representations of the welding process and to evaluate their relative contributions using multiple feature selection techniques. Therefore, the reported feature importance should be interpreted as reflecting the collective influence of correlated process descriptors rather than completely independent physical effects.

### 3.4 Machine learning model performance and hyperparameter optimization results

Since the experimental dataset only had 25 samples, LOOCV was utilized to maximize the use of the available data and minimize the chance of overfitting. Every single observation in the experiment was validated once, while the other samples were used for training and the results yield reliable evaluations for small sample sizes. Moreover, hyperparameter tuning, residuals analysis, and SHAP-based interpretability techniques were implemented to ensure that the created models capture physical meaningful relationships and do not overfit on the experiment data.

LOOCV ensures that most of the available data is used by running many iterations with 24 cases being used to train the model and 1 sample being used for testing. Using LOOCV for validation provides an internal estimate of model performance on small datasets.

The performance of six contemporary regression algorithms that were trained with physics-based features was measured including Extreme Gradient Boosting (XGBoost), Gaussian Process Regression (GPR), Random Forest (RF), CatBoost, Support Vector Regression (SVR), and Light Gradient Boosting Machine (LightGBM). The evaluation measures included Mean Absolute Error (MAE), Root Mean Square Error (RMSE), Coefficient of Determination (R^2^), Mean Absolute Percentage Error (MAPE), and Adjusted R^2^. Additionally, Optuna-based hyperparameter optimization was performed for the algorithms ahead of the final evaluation process involving LOOCV.

The close agreement in tensile strength results between experimental and predicted values, the distribution of residuals around 0, absence of systematic prediction bias, and consistent results of SHAP-based feature importance indicate that the proposed method captures meaningful relations.

The graph in Figure 8 illustrates a comparison of the Root Mean Square Error (RMSE) values employed by six regression techniques during LOOCV. Since the RMSE provides a higher penalty for large prediction errors compared to MAE, lower the RMSE, the better the model. According to the data obtained, XGBoost showed the minimum RMSE value of 9.88 MPa and thus demonstrated the best forecasting capabilities. Gaussian Process Regression and Random Forest provided relatively low RMSE values equal to 11.60 MPa and 12.58 MPa, respectively.

**Figure 8:**
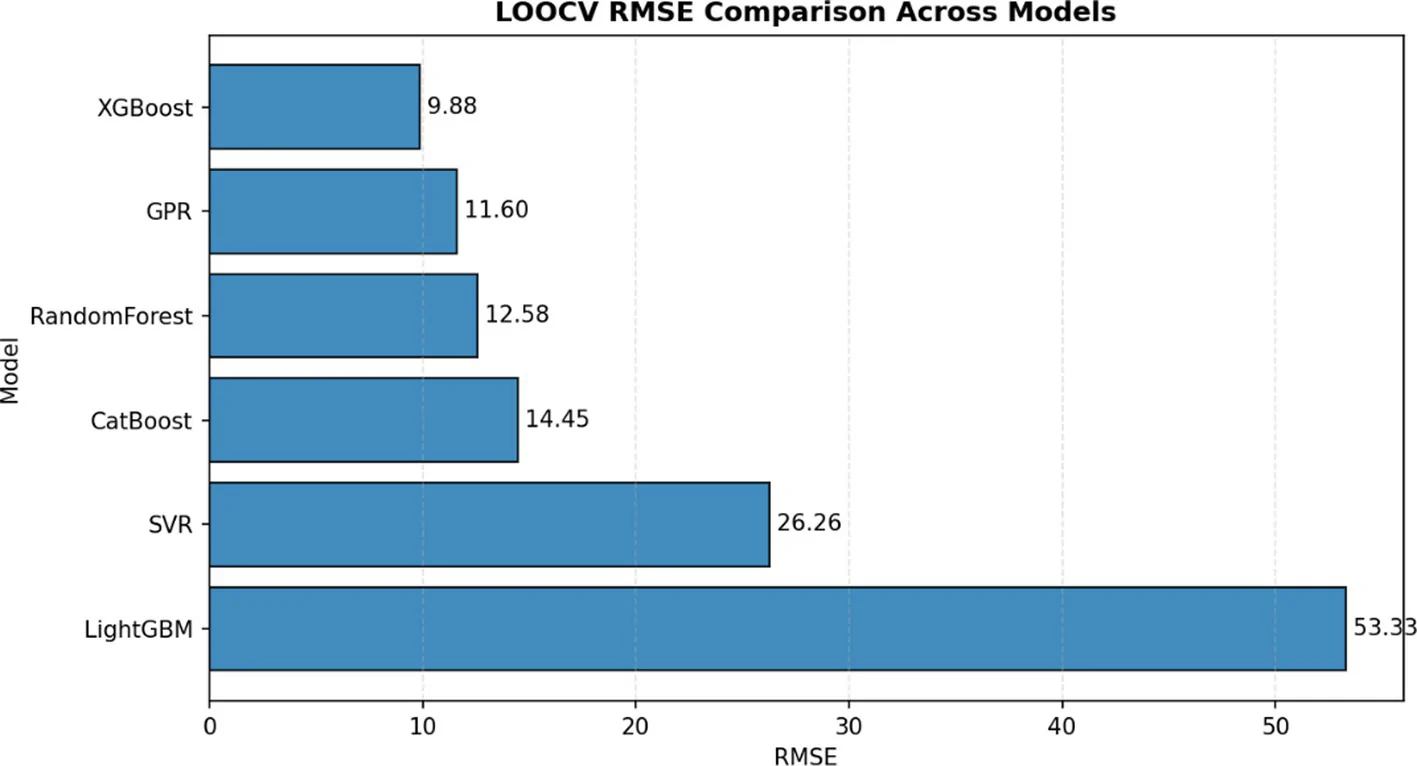
LOOCV RMSE comparison across machine learning models.

Hence, these techniques demonstrated the ability to address the nonlinear relationships between the input parameters and the tensile strength. CatBoost showed moderate prediction performance, as its RMSE value was 14.45 MPa. On the other hand, Support Vector Regression exhibited much worse results with RMSE equal to 26.26 MPa. LightGBM showed the worst performance providing an RMSE of more than 53 MPa. The poor performance can be explained by the very small sample that LightGBM algorithms were based on meaning that the method had some difficulties while creating boosting trees that would not be prone to overfitting. Figure 8 demonstrates that ensemble boosting approaches, particularly XGBoost, provide superior prediction capability for small experimental datasets combined with physics inspired engineered features.

Figure 9 shows the connection between tensile strength data obtained from experiments and calculated from the enhanced XGBoost model during LOOCV. The dashed line represents the ideal condition in which predicted values correspond to observed value. The points showing predictions are gathered near the ideal line, which indicates a good correspondence between real figures and predicted values. Some disagreements are only observed at the low and high ends of the tensile strength range proving that strength prediction accuracy is steady throughout the range of experimental values.

**Figure 9:**
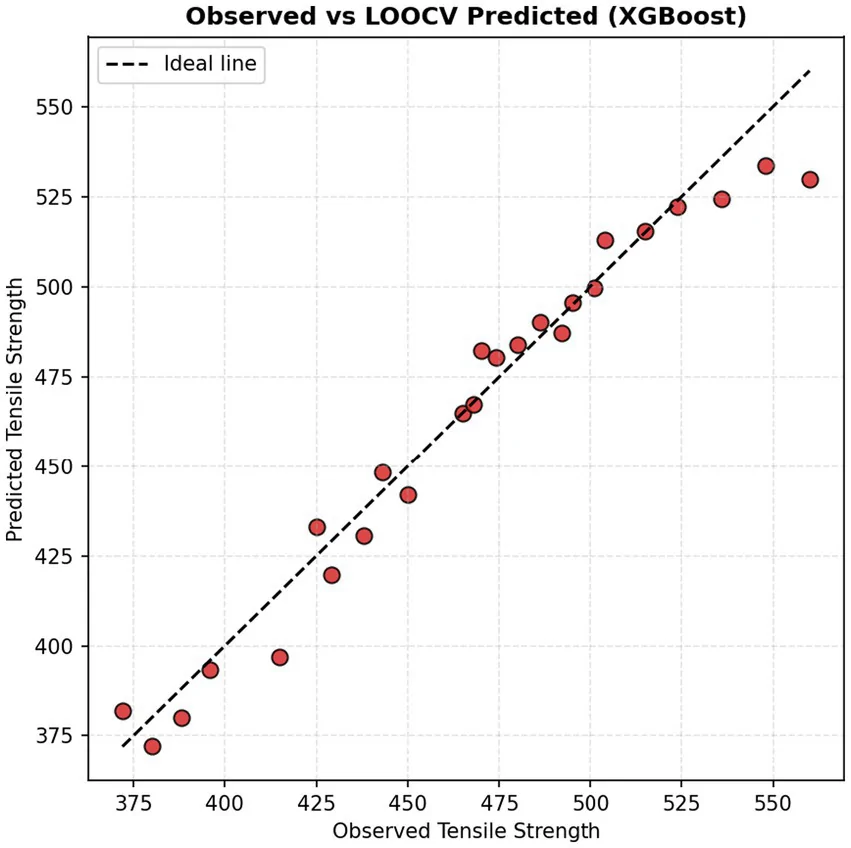
Observed vs. LOOCV predicted tensile strength using the XGBoost model.

The fact that the points related to predictions are gathered close to the ideal line indicates that the model can predict tensile strength correctly and captures nonlinear relationships between the reinforcement type, processing parameters and the engineered physics inspired descriptors. Additionally, the absence of systematic overestimation shows that the model does not have any important bias and it is effective as demonstrated during the LOOCV validation. Thus, Figure 9 demonstrates that the XGBoost model is valid for use in forecasting the tensile strength of CNT-Si₃N₄ reinforced FWTPET joints.

Table 20 displays the results of the predictive performance of all machine learning models, measured using five widely used regression metrics: MAE, RMSE, R^2^, Adjusted R^2^, and MAPE. These metrics jointly assess prediction accuracy, error size, goodness-of-fit, model complexity, and relative prediction error.

**Table 20:** Performance comparison of machine learning models using leave-one-out cross validation.

Model	MAE (MPa)	RMSE (MPa)	R^2^	Adjusted R^2^	MAPE (%)
XGBoost	7.4531	9.8824	0.9627	0.9361	1.6051
GPR	9.0742	11.6049	0.9486	0.9119	1.9459
Random Forest	8.3332	12.5776	0.9396	0.8965	1.8121
CatBoost	9.5767	14.4535	0.9203	0.8634	2.0320
SVR	19.1274	26.2614	0.7369	0.5489	4.1336
LightGBM	43.8967	53.3298	−0.0851	−0.8601	9.6930

Table 21 presents the optimal hyperparameter settings discovered with the help of the Optuna optimization framework, where the optimization procedure minimized the LOOCV RMSE by automatically searching the hyperparameter search space for each of the regression algorithms. The optimized parameters were used to train the final predictive models after that.

**Table 21:** Optuna-optimized hyperparameters for machine learning models.

Model	Best LOOCV RMSE	Best hyperparameters
XGBoost	5.7001	n_estimators = 737, max_depth = 3, optimized learning rate and regularization parameters
LightGBM	7.1666	n_estimators = 559, max_depth = 5, optimized learning rate and boosting parameters
CatBoost	7.7115	iterations = 472, depth = 5, optimized learning rate
Random Forest	7.9540	n_estimators = 334, max_depth = 9, optimized minimum sample parameters
GPR	9.0712	Optimized Constant Kernel and RBF kernel parameters
SVR	15.6097	RBF kernel with optimized C and ε parameters

For consistency, the performance values reported as the final model results correspond to the hyperparameter-optimized models evaluated using LOOCV. Results obtained during preliminary model development or before optimization are not presented as final performance values. Similarly, SHAP-based feature importance values are explicitly associated with the final selected XGBoost model and its corresponding feature set. Feature-selection results obtained using RFE, Boruta, permutation importance, and SHAP are presented separately as complementary analyses and are not interpreted as equivalent performance measures. This distinction ensures that model performance, feature ranking, and explainability results are traceable to their respective analytical configurations.

Table 22 shows the performance of the optimized regression models determined by LOOCV after hyper-parameter tuning. The optimized XGBoost model performed the best overall prediction with the minimum MAE and RMSE values, and the maximum R^2^ and Adjusted R^2^ values. The rest of the ensemble based models also performed well, while the SVR model had relatively higher prediction errors.

**Table 22:** Final performance of optimized machine learning models.

Model	MAE	RMSE	R^2^	MAPE (%)	Adjusted R^2^
XGBoost	5.7001	7.7380	0.9772	1.2590	0.9608
LightGBM	7.1666	10.2066	0.9603	1.5188	0.9319
GPR	9.3831	11.8347	0.9466	2.0059	0.9084
CatBoost	7.7115	12.0454	0.9446	1.6794	0.9051
Random Forest	7.9540	12.1081	0.9441	1.7065	0.9041
SVR	15.6097	22.2605	0.8109	3.2578	0.6759

The comparative analysis shows that XGBoost performed best among the evaluated models. It exhibited the lowest values of MAE (5.70), RMSE (7.74), highest R² (0.9772) and Adjusted R^2^ (0.9608), and the lowest MAPE (1.26%) performance, indicating high prediction capability and good generalization performance under LOOCV.

The gradient boosting strategy of XGBoost is the main reason for its superior performance, as it sequentially corrects the error of prediction, and successfully models the complex nonlinear relationship between the process parameters and tensile strength. Moreover, XGBoost has regularization features which prevent overfitting, making it well suited for small experimental datasets, especially the 25 FWTPET observations utilized in this research.

Similarly, LightGBM also performed very well after Optuna optimization, with its RMSE dropping from ~53 MPa before the optimization to 10.21 MPa after it. This significant enhancement demonstrates the significance of hyperparameter optimization in boosting algorithms. The prediction accuracy of the GPR, RF and CatBoost models were found to be similar, suggesting that they can also model the non-linear behavior of the FWTPET process.

The SVR always generated higher prediction errors than the ensemble based methods. While SVR can model nonlinear functions, its performance is sensitive to the choice of kernel and the tuning of the kernel parameters. Ensemble learning was found to be more effective at representing the complex interactions of reinforcement composition, heat generation, and material flow in the present research.

The overall results showed that physics inspired engineered features and Optuna-optimized XGBoost offered the highest accuracy and reliability framework for tensile strength prediction. The good prediction accuracy and good agreement between observed and predicted values validate the efficiency of the suggested machine-learning approach to the FWTPET process.

The physics inspired machine learning performance evaluation shows that the proposed framework for physics inspired machine learning can predict the tensile strength of CNT–Si₃N₄ reinforced FWTPET joints with high accuracy. LOOCV enabled a statistically sound evaluation for the small experimental set and Optuna Hyperparameter Optimization further improved the model performance.

In comparison to all algorithms considered, XGBoost turned out to be the best model with minimum prediction errors and maximum goodness of fit. Through the observed-versus-predicted analysis, it was concluded that the excellent agreement between the observed data and the predicted data showed that there was not much bias in the prediction and the generalization ability was good. The results also demonstrate that proper integration of physics inspired feature engineering, cross validation and advanced ensemble learning techniques can provide reliable, accurate and interpretable prediction of tensile strength, giving a solid foundation to the subsequent XAI–SHAP analysis.

In order to assess the performance of the suggested physics-based framework, several models serving as baselines were constructed based on linear regression with CNT, polynomial regression with CNT, and several machine learning techniques that only rely on original variables of the process like CNT, Si₃N₄, rotation speed, and plunge depth, under the same protocol of LOOCV and their results were compared with those of the present framework.

Table 23 presents the analysis of different ML models regarding their performance measured in terms of RMSE and R^2^ which results indicate that its suggested XGBoost model, equipped with physics-based features, provides the best results when it comes to both RMSE (7.7380) and R^2^ (0.9772), that proves that the use of physics-based features leads to improved performance in comparison with conventional Linear Regression, Polynomial Regression, and XGBoost approaches that rely on original variables.

**Table 23:** Feature and CNT performance comparison of machine learning models.

Model	Input features	RMSE	R^2^
Linear Regression	CNT	13.6820	0.9286
Polynomial Regression (Degree 2)	CNT	14.1394	0.9237
Polynomial Regression (Degree 3)	CNT	13.8568	0.9267
XGBoost	Original 4 features	11.5950	0.9487
Proposed XGBoost	Physics inspired features	7.7380	0.9772

An ablation study was conducted to measure the effects of the proposed physics inspired feature engineering framework. Four different model configurations were tested with the same LOOCV protocol: (i) CNT-only configuration, (ii) the original experimental features (CNT, Si₃N₄, RPM, and plunge depth), (iii) physics inspired engineered features only, and (iv) the combination of both original and physics inspired features set. The results show that introducing physics inspired descriptors improves the predictive performance considerably.

The ablation study in Table 24 shows the impact of introducing the proposed physics informed feature engineering method. This evaluation was based on four variations of the input feature set (CNT, Si₃N₄, RPM, and Depth) using the LOOCV approach. The original features set was the most successful with respect to the predictions made. The lowest MAE (8.3544 MPa), RMSE (11.5950 MPa), and MAPE (1.8137%) results along with the highest R^2^ value (R^2^ = 0.9487) were attained. The tested CNT model gave reasonable but poorer results compared to the model where both CNT and original parameters were applied. Using only the physics informed features resulted in the least accurate predictions, which suggests that these features cannot substitute for the original experimental variables. The results obtained show that the resulting model prediction accuracy gives good results but does not improve when predictions are based on using the combination of original and physics inspired features.

**Table 24:** Ablation study of the proposed physics inspired framework.

Configuration	MAE	RMSE	R^2^	MAPE (%)
CNT Only	11.0400	14.0232	0.9250	2.4014
Original Features (CNT, Si₃N₄, RPM, Depth)	8.3544	11.5950	0.9487	1.8137
Physics Features Only	18.5303	22.7199	0.8031	3.8893
Original + Physics Features	9.9977	13.5174	0.9303	2.1718

### 3.5 Explainable artificial intelligence analysis

Despite the successful performance of the XGBoost model in the predictive tasks, being able to explain the reasons behind the given prediction is just as paramount considering scientific interpretation and practical decisions. As a result, XAI built on SHAP was used to explain how the original process parameters and proposed physics inspired engineered parameters affect tensile strength predictions.

The SHAP framework assigns a specific contribution, referred to as Shapley value from cooperative game theory to every model feature when it comes to formulating the final prediction. Unlike classical feature importance assessments, SHAP does allow identifying the most significant features and understanding whether they impact the predicted value in a positive or negative manner. Given that XGBoost has shown the maximum predictive performance it was chosen as the selected model for the SHAP analysis. In total, 10 features have been analyzed: 2 experimental variables and 8 physics inspired variables, which makes things clearer from the point of view of the underlying processes at work in the FWTPET process.

The SHAP summary plot, illustrated in Figure 10, generates a global explanation about importance of features regarding all experiments. The horizontal axis shows the SHAP values of features, which suggest how much and in what way certain features influence the prediction of tensile strength. In other words, positive values of SHAP means increase of the predicted tensile strength, while negative values indicate a decrease in this prediction. The color scale shows the size of the feature value: blue means small values and red means high values.

**Figure 10:**
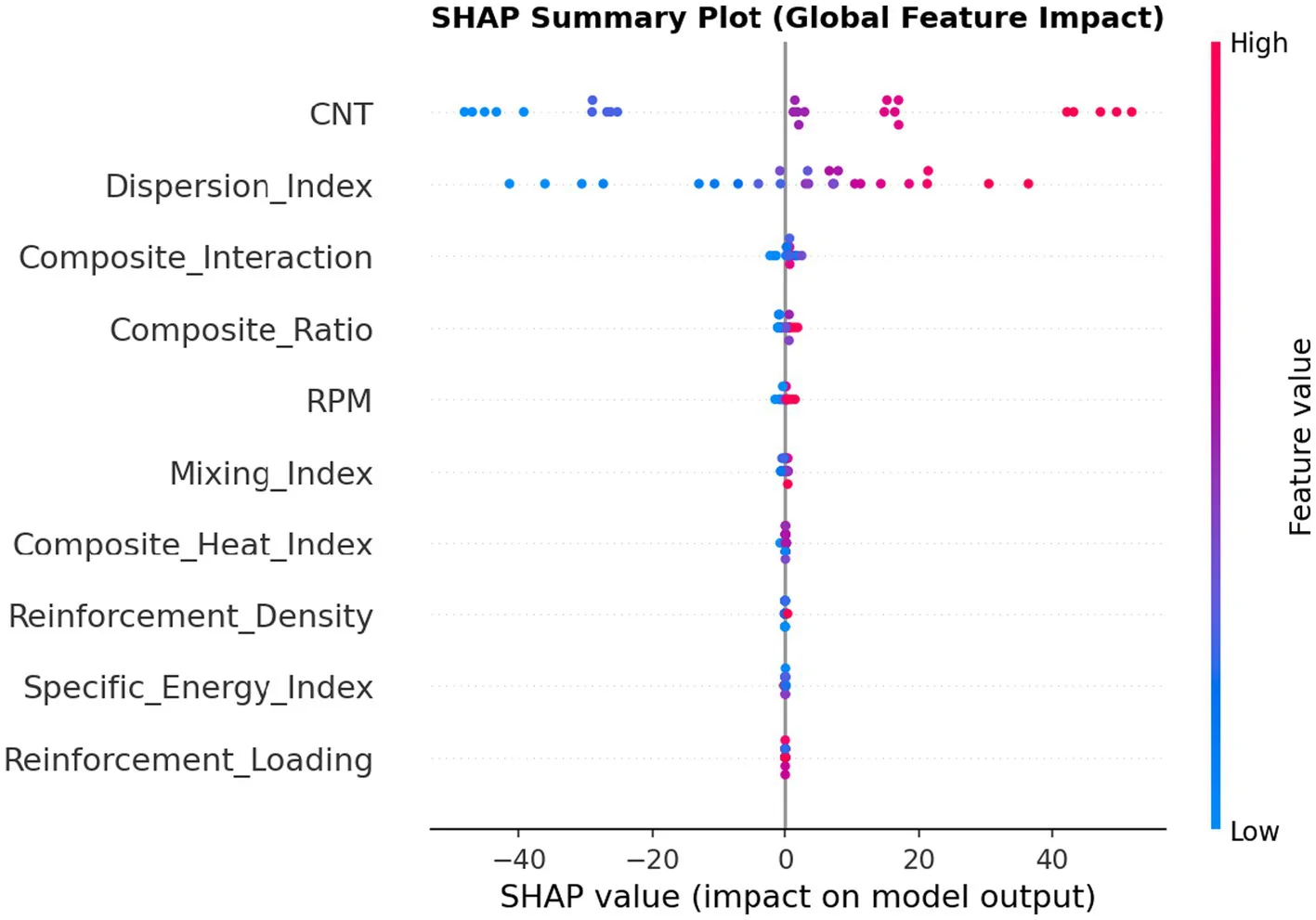
SHAP summary plot showing the global influence of original and physics inspired features.

It is obvious from the figure that CNT is the most important feature, since it possesses the broadest scope of SHAP values from around −50 MPa to +52 MPa. The points on the plot connected with high values of CNT (in red) are in the range of positive SHAP values indicating that high concentration of CNT contributes positively to increase in tensile strength. On the contrary, points corresponding to low CNT concentration (in blue) affect negatively the prediction owing to low effectiveness of reinforcement established by low value of CNT.

The second descriptor with the most impact is the Dispersion Index which gives rise to positive values of SHAP. This implies that maximizing the use of dispersion leverages proper tool speed to help in transfer of load and production of a more uniform microstructure of welds. Other physics-based descriptors such as Composite Interaction, Composite Ratio, Mixing Index, Composite Heat Index, Reinforcement Density, Specific Energy Index, and Reinforcement Loading have smaller values of SHAP but help in refining the predictions of the model. While the predicted results are mainly defined by CNT and the dispersion of reinforcement, other engineering descriptors account for some secondary influences related to thermo-mechanical aspects of tensile strength.

The picture presented in Figure 11 shows the global importance of various features determined with the help of the mean SHAP value. The larger bars represent those features which play a greater role in all the experiments. The CNT feature has the maximum mean absolute SHAP value equal to 27.30, which is significantly greater than that of the other features. The Dispersion Index comes second with the mean SHAP value of 14.98, which means that reinforcement dispersion is the second mechanism that greatly affects the tensile strength. The other features are Composite Interaction and Composite Ratio, which have lower SHAP values compared to the previous features and confirm that the synergy provided by hybrid reinforcement helps predict tensile strength not to such an extent as CNT content and reinforcement dispersion mechanisms.

**Figure 11:**
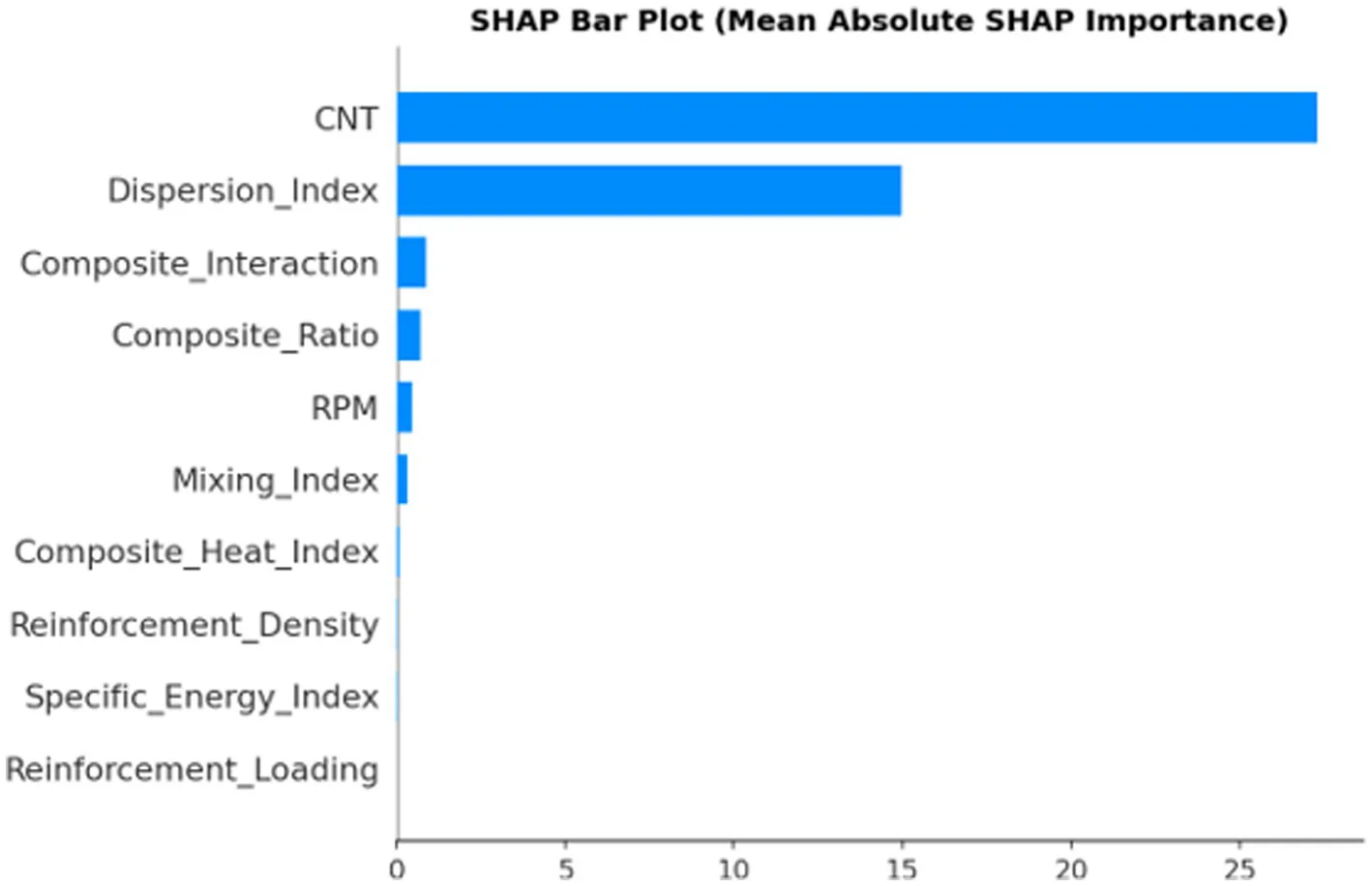
Mean absolute SHAP feature importance ranking.

All other features such as RPM, Mixing Index, Composite Heat Index, Reinforcement Density, Specific Energy Index, and Reinforcement Loading have rather low SHAP values. This indicates that the variables are globally significant and primarily contribute to improving local predictions, rather than making predictions in general. The ranking based on SHAP indicates that the physics inspired variables achieve their goal of capturing meaningful mechanisms while being easily explainable.

In Figure 12, the SHAP dependence plot for the Dispersion Index is displayed, with the horizontal axis showing Dispersion Index values and the vertical axis showing the SHAP effects on the calculations of tensile strength. The color scheme displays the Composite Ratio, which indicates the interaction of the features. There is a visible positive relationship between the Dispersion Index values and the obtained SHAP values. As the values of the Dispersion Index go up, the results become more positive, showing the relationship between the reinforcement dispersion and predicted tensile strength. Also, the gradient of the colors indicates that there is interaction between the Dispersion Index values and the Composite ratio. Values of the Composite Ratio closer to the highest show high SHAP effects, meaning that the effect of the reinforcement dispersion is increasing along with the increase of CNT content.

**Figure 12:**
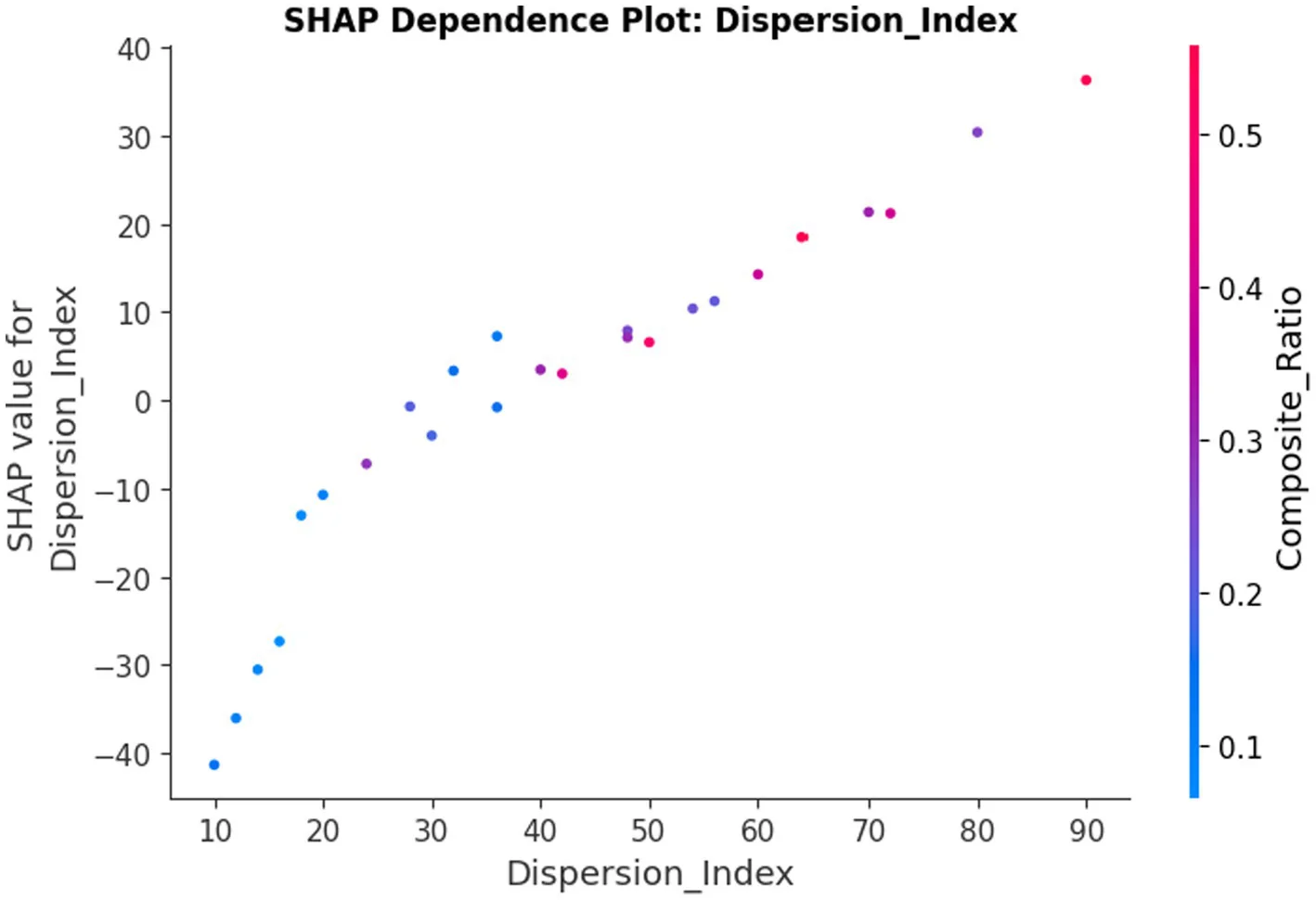
SHAP dependence plot for the dispersion index.

The SHAP waterfall plot is shown in Figure 13 explaining how the prediction is arrived at for a sample experimental case. The waterfall plot shows the contribution of each feature in shifting the prediction from the baseline value of the model to the predicted value. The prediction starts with the baseline value of about 466 MPa which corresponds to the mean tensile strength of the test samples. Afterwards, the contribution of each of the features changes the baseline value in some manner. The contribution of the CNT is the greatest and provides nearly +51.8 MPa of change, while the Dispersion Index provides +36.35 MPa. Other functions such as Composite Interaction, Composite Ratio, RPM, and Mixing Index provide a smaller but still positive change.

**Figure 13:**
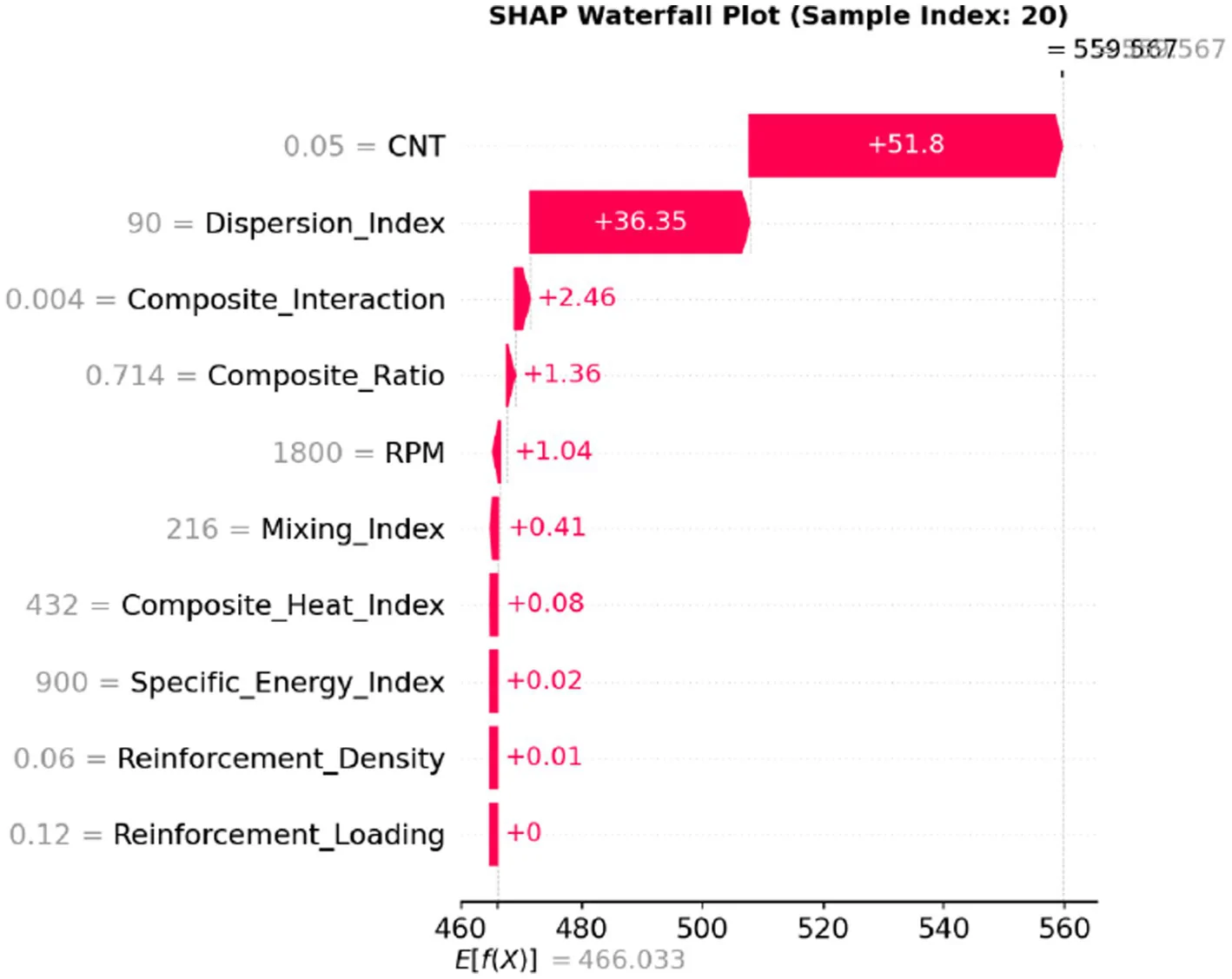
SHAP waterfall plot explaining an individual prediction.

The waterfall image demonstrates that the tensile strength is mostly dependent on the characteristics of the reinforcement and its dispersal rather than the parameters of the production process. This finding proves the justification of the physical explanations made on the ground of the global SHAP analysis.

Table 25 summarizes the global feature importance obtained using the mean absolute SHAP values. Larger SHAP values indicate greater average influence on tensile strength prediction across all experimental observations.

**Table 25:** Global SHAP feature importance ranking.

Rank	Feature	Mean absolute SHAP
1	CNT	27.300659
2	Dispersion Index	14.983090
3	Composite Interaction	0.881960
4	Composite Ratio	0.700490
5	RPM	0.455980
6	Mixing Index	0.322220
7	Composite Heat Index	0.088550
8	Reinforcement Density	0.046820
9	Specific Energy Index	0.036510
10	Reinforcement Loading	0.000240

Table 26 shows the SHAP ranking of the importance of the physics inspired engineering descriptors. The results show that reinforcement dispersion is significantly more prominent than the engineered, which is necessary to the friction welding process for the dispersion index. SHAP interpretation allows for the understanding of the machine learning model trained on the data and its use of different input features to predict tensile strength. In fact, SHAP does not make direct statements on causal relationships and does not prove the physical mechanisms involved in the welding process. Instead, the feature contributions identified represent the relative significance of each descriptor within the predictive model. Each of these descriptors has a physical meaning, which is interpreted individually in light of existing theory on friction welding and materials science.

**Table 26:** SHAP ranking of physics inspired engineered features.

Rank	Physics inspired feature	Mean absolute SHAP
1	Dispersion Index	14.983090
2	Composite Interaction	0.881960
3	Composite Ratio	0.700490
4	Mixing Index	0.322220
5	Composite Heat Index	0.088550
6	Reinforcement Density	0.046820
7	Specific Energy Index	0.036510
8	Reinforcement Loading	0.000240

### 3.6 Physical interpretation and engineering implications of SHAP analysis

The SHAP analysis offers a clear understanding of the optimized XGBoost model, by revealing the impact of each feature on the prediction of tensile strength. The global SHAP summary plot and the SHAP bar plot show that the most important predictor is CNT, which is consistent across both types of plots and indicates that the concentration of CNT is the most important variable in the mechanical performance of the FWTPET joints. The positive SHAP values with high content of CNTs suggest improved load transfer, grain refinement and crack bridging in the weld area.

The Dispersion Index has the greatest importance among the engineered descriptors, indicating that a uniform distribution of CNT particles is crucial to achieve high tensile strength under proper tool rotational conditions. The effects of hybrid reinforcement are observed as composite interaction and composite ratio, which further indicate that hybrid reinforcement has a positive reinforcement effect, promoting synergistic reinforcement among CNT and Si₃N₄ particles. The dependence plot further confirms that the values of SHAP increase almost in a linear fashion as the Dispersion Index increases, showing that mechanical performance improves with the improvement of reinforcement dispersion.

The waterfall plot further highlights that the prediction of a given sample is not due to one dominant variable but rather is the result of the sum of various physically meaningful descriptors. The major and positive values of CNT and Dispersion Index, along with a small contribution from the other engineered descriptors, prove that the proposed physics inspired descriptors are effective in capturing the coupled effects of reinforcement loading, heat generation, material flow and plastic deformation throughout the FWTPET process.

In summary, the proposed physics inspired feature engineering approach is scientifically relevant, as indicated by the SHAP analysis. The XAI framework not only clarifies the model’s predictions of tensile strength but also offers insights into how the processing variables contribute to its enhancement.

The XAI analysis shows that the optimized XGBoost model is accurate and interpretable. The most important predictors according to SHAP analysis were the reinforcement content and the Dispersion Index, meaning that the reinforcement content and the dispersion of reinforcements are the most important mechanisms responsible for tensile strength. Other engineered descriptors gave rise to smaller but more physically relevant effects for reinforcement interaction, reinforcement mixing, heat generation and reinforcement distribution.

The physics inspired machine learning framework proposed in this work shows interpretability without compromising on capturing the thermo-mechanical behavior of the FWTPET process as demonstrated by the SHAP summary plot, feature importance ranking, dependence plot and waterfall explanation. This result supports the use of physics inspired feature engineering together with Explainable Artificial Intelligence as a powerful technique for building scientifically informed and trustworthy predictive models for complex friction welding processes.

### 3.7 Residual and reliability analysis

After assessing how well machine learning algorithms performed, it was decided to perform a residual and reliability analysis to study the robustness and stability of the physics inspired XGBoost framework. It is widely known that while the performance indicators RMSE, MAE, and R^2^ quantify the degree of accuracy of predictions, it is not the basis for assessing whether a prediction is systematically biased or happens in a random way. To check how reliable the established model is, residual analysis, the method of prediction versus actual comparison and learning curve analysis were used.

Residual graphs were created to examine distribution of prediction errors and the actual versus predicted graph was employed to check if experimental and model tensile strength values coincide. The learning curve was used to find out whether the model experienced the issues of underfitting or overfitting and analyze its learning process as new data appeared. The results of these analyses provide enough evidence that the developed physics-based machine learning framework can predict tensile strength of FWTPET joints.

Table 27 presents the predictive capabilities of various ML models assessed with the LOOCV methodology. This analysis considered all performance measures, including the MAE, RMSE, R^2^, MAPE, adjusted R^2^, and the total rank of the models. In general, lower values of MAE, RMSE, and MAPE and higher R^2^ correspond to better performance and predictive accuracy.

**Table 27:** Performance comparison of machine learning models.

Final rank	Model	MAE	RMSE	R^2^	MAPE (%)	Adjusted R^2^	Composite rank score
1	XGBoost	5.7001	7.7380	0.9772	1.2590	0.9608	1.000
2	LightGBM	7.1666	10.2066	0.9603	1.5188	0.9319	2.000
3	CatBoost	7.7115	12.0454	0.9446	1.6794	0.9051	3.600
4	GPR	9.3831	11.8347	0.9466	2.0059	0.9084	3.800
5	Random Forest	7.9540	12.1081	0.9441	1.7065	0.9041	4.600
6	SVR	15.6097	22.2605	0.8109	3.2578	0.6759	6.000

From the above analysis, XGBoost outperformed all other types of regression methods. The model attained the lowest MAE value of 5.70, the lowest RMSE value of 7.74, and the highest R^2^ value of 0.9772, which indicates a great agreement between its predictions and the experimental data on tensile strength. In addition, the very low MAPE value of 1.26% confirms that the prediction errors are minimal when compared to the actual results.

The second position is held by LightGBM, which has yielded a satisfactory performance but has a higher prediction error than XGBoost. CatBoost, Gaussian Process Regression, and Random Forest showed rather similar results and a medium level of prediction accuracy. Support Vector Regression showed the worst result among all approaches due to the high value of RMSE and the low value of R^2^.

XGBoost’s success can be mainly found in its gradient boosting approach that allows modeling of nonlinear interactions between the actual process variables, and the suggested physics inspired engineered feature set. Its ensemble learning technique makes it possible to reduce prediction errors while remaining able to generalize well even with a small experimental dataset available.

Figure 14 shows the residual distribution derived from the optimized XGBoost model using LOOCV technique. The residuals are defined as the difference between the experimentally obtained tensile strength value and the predicted value. The residuals can be randomly distributed along the zero-error line without indicating any systematic pattern. Both positive and negative residuals exist in the range of predictions indicating that the model does not always predict tensile strength either too high or too low.

**Figure 14:**
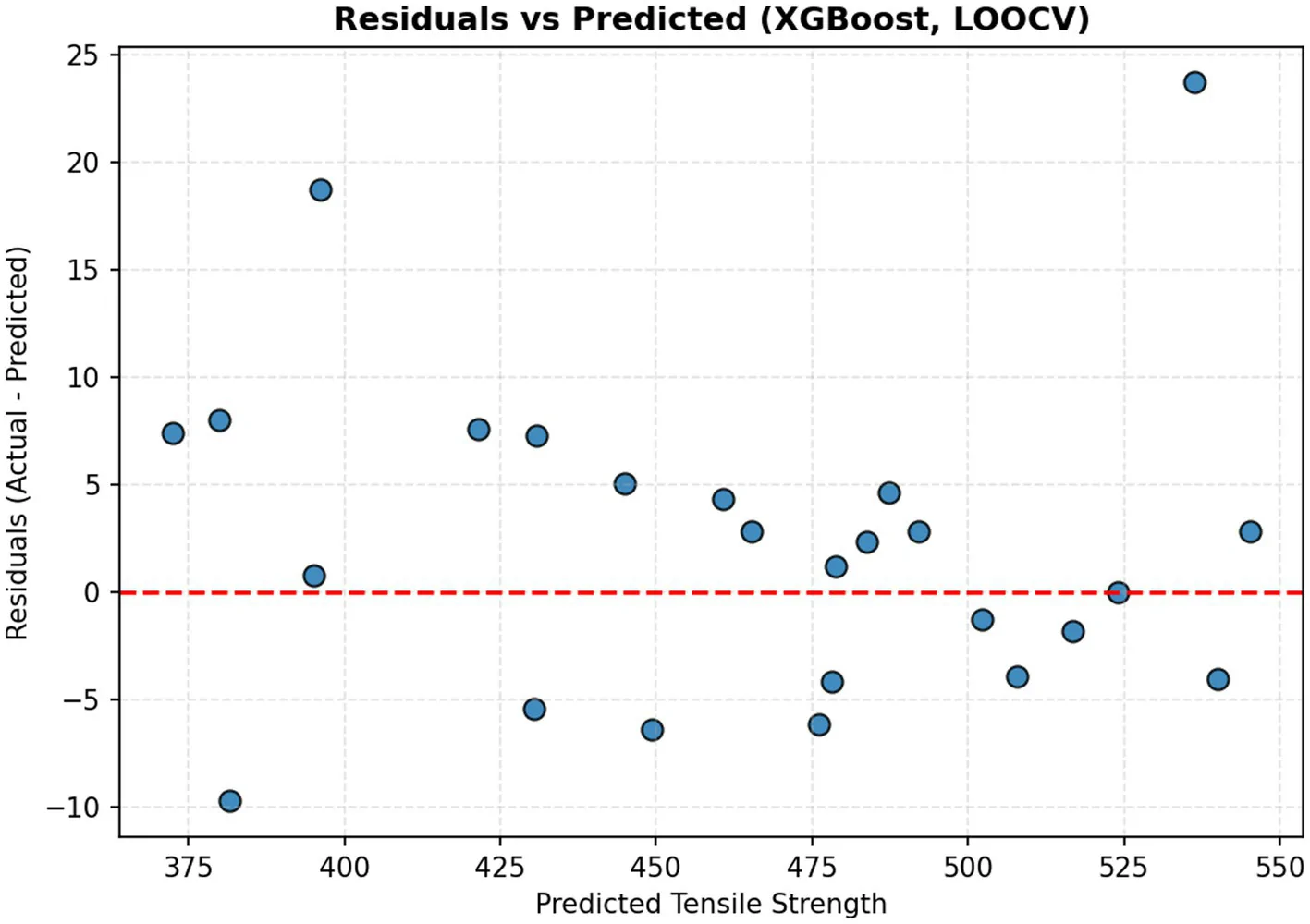
Residuals vs. predicted tensile strength for the XGBoost model.

Most of the residual values come into the range of ±8 MPa, and there are only few of the residuals observed to deviate from this limit. In addition, the variance of residuals remains unchanged throughout the range thereby showing the absence of heteroscedasticity. The randomness of residuals provides evidence that the prediction errors are mostly random and not systematic which means that the XGBoost model developed can adequately capture the relation between process parameters and tensile strength.

The graph in Figure 15 indicates a comparison between experimental tensile strengths and the predicted tensile strengths by the optimized XGBoost model. The diagonal dash line in the graph is the ideal prediction line where predicted tensile strength value is exactly equal to measured tensile strength value. In this figure, most of the data points are located near to the ideal line indicating close agreement between predicted and experimental tensile strengths. Only a few data points deviate from the ideal line, and they do so in the extreme ranges of tensile strength values. The proximity of majority of the data around the ideal line shows the high accuracy of the predicted data. The high similarity of the predicted values and experimental values in this case proves that the suggested physics inspired feature engineering technique can catch the main physical phenomena affecting the FWTPET process.

**Figure 15:**
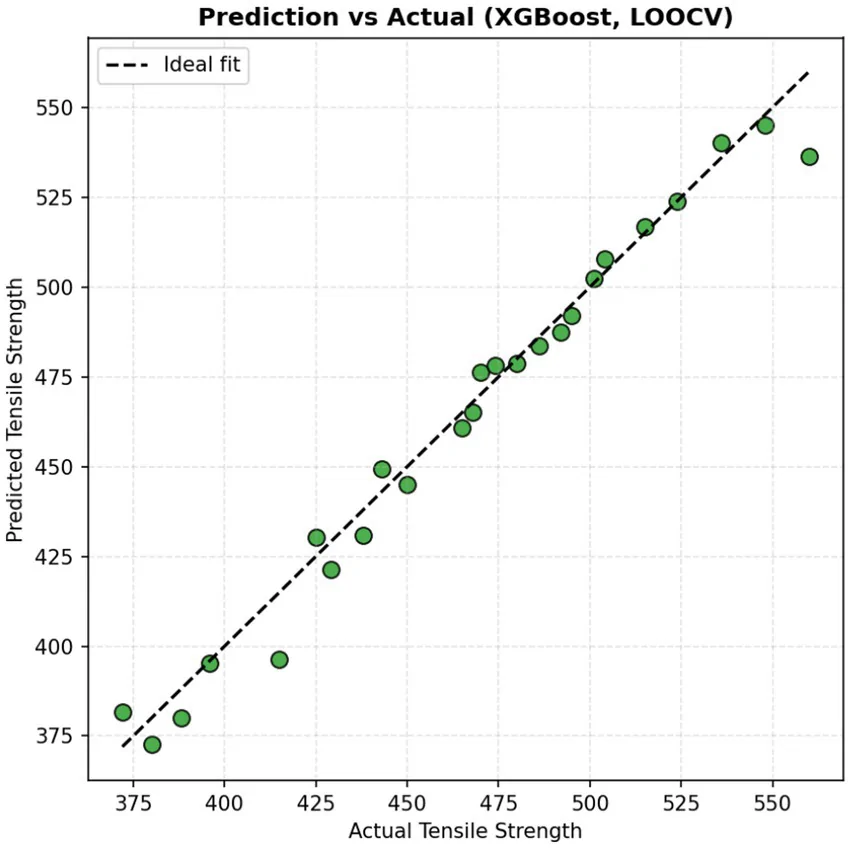
Comparison of experimental and predicted tensile strength.

Figure 16 illustrates the learning curve for the optimized XGBoost model. The blue curve is the RMSE of the training set while the red curve is the RMSE of the validation set as a function of the size of the training set.

**Figure 16:**
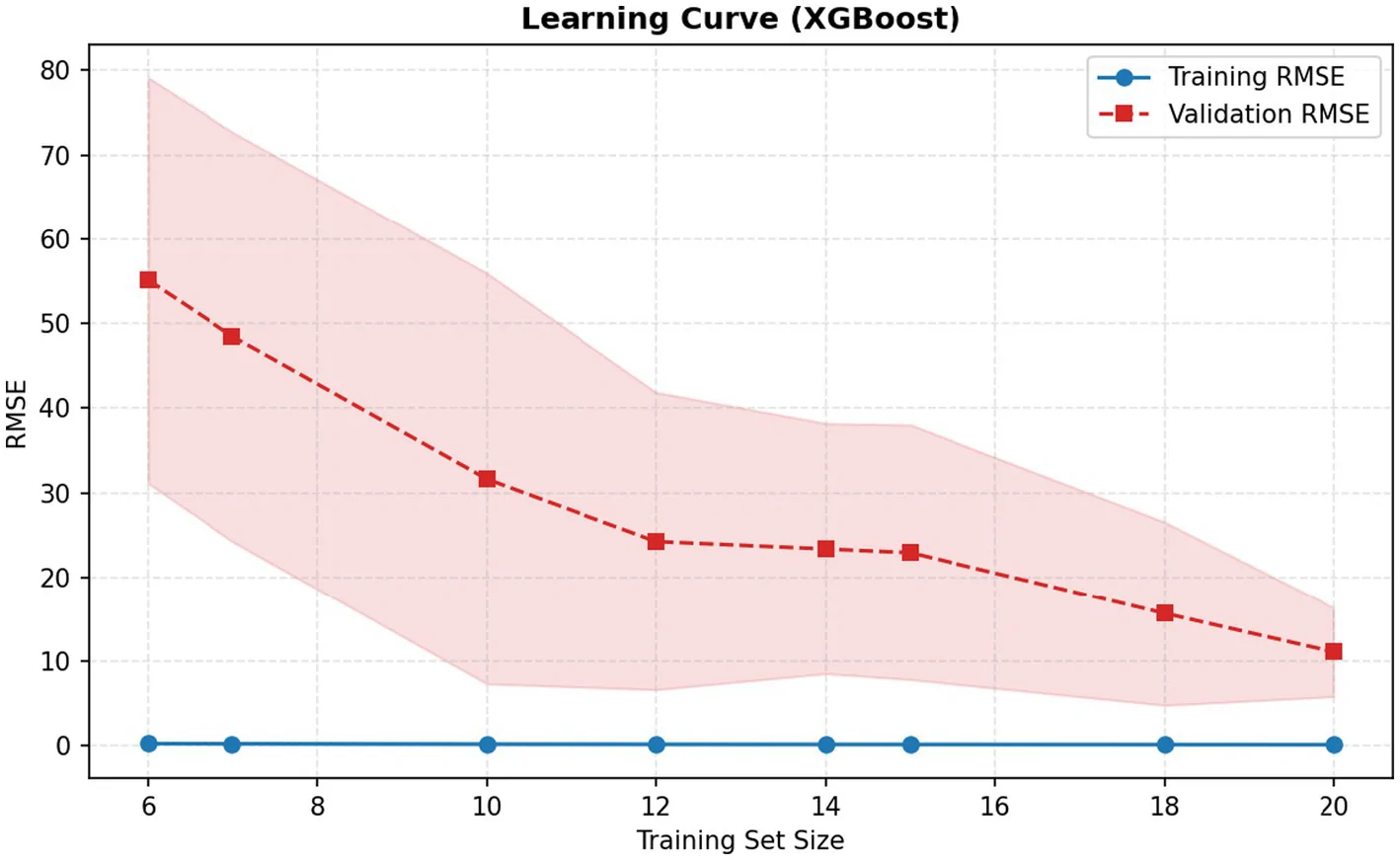
Learning curve of the optimized XGBoost model.

The model consistently learns the provided experimental data, as indicated by the small training RMSE across all training sizes. More importantly, the validation RMSE shows a progressive downward improvement as more samples are added to the training set, ensuring improvement in prediction accuracy. The decrease in the validation error indicates that there is no serious overfitting of the model, yet it still gains from more experimental data.

The gap between training and validation errors is caused by the small size of the data set, but the stochastic error of the validation error decreases with the increase of the training set, indicating that the learning behavior is good and that the model has good generalization capability. The learning curve also suggests that more experimental results should be available to further enhance prediction performance while retaining stability of the model.

The residual and reliability analyses clearly show that the proposed physics inspired XGBoost model can accurately and reliably predict the tensile strength. The residual plot also shows that the prediction errors are randomly distributed about zero, suggesting no systematic error in the model’s predictions for various tensile strength levels.

Likewise, the agreement between the experimental and predicted results shows very good agreement between the two. The near perfect agreement of most data points to the ideal prediction line supports the high R^2^ value that was found during the quantitative model evaluation, and the optimized XGBoost model can capture the non-linear correlation between the process parameters, engineered descriptors and tensile strength.

The learning curve also reinforces the strong foundation of the suggested framework. As the number of training cases increases, the model’s RMSE for the validation set decreases, which shows that the model can learn from the limited experimental data it has while showing a stable prediction performance. The small difference between the training and validation errors is expected due to the few experimental samples but no sign of bad overfitting or underfitting is found. This means that the inclusion of physics inspired engineered features leads to better generalization of the model by considering physically relevant information as opposed to solely statistical correlations.

In conclusion, the residual analysis, prediction comparison, and learning behavior all agree that the proposed machine learning framework can make accurate, unbiased, and stable predictions that are suitable for engineering applications of friction welding of aluminum alloy tube-to-tube plate joints.

The residual analysis and reliability analysis further validate the proposed physics inspired XGBoost framework for its robustness and applicability. XGBoost model had the highest coefficient of determination (R^2^ = 0.9772) and the lowest MAE (5.70) and RMSE (7.74) as well as the best composite ranking score (1.00) among all the machine learning models evaluated. The residual plot showed the errors of prediction were randomly distributed around zero, meaning that there was no systematic pattern and that the model was behaving well. The experimental results were very well matched with the actual results and the learning curve indicated stable convergence and adequate generalization performance under LOOCV.

The results validate the use of the physics inspired feature engineering to enhance the XGBoost algorithm, which leads to a high-level reliable predictive model that can capture the intricate thermo mechanical phenomena involved in FWTPET. As a result, the suggested methodology is a powerful and meaningful tool for predicting tensile strength and less experimentation to optimize the process in advanced friction welding applications.

Although LOOCV provides an appropriate validation strategy for small experimental datasets, feature selection and Optuna-based hyperparameter optimization were not implemented as fully nested procedures within each cross-validation training fold. Consequently, a certain degree of optimistic bias in the reported performance metrics cannot be completely excluded. Therefore, the predictive performance presented in this research should be interpreted as a proof-of-concept demonstrating the potential of the proposed physics-inspired machine learning framework. Future investigations will incorporate nested cross-validation together with larger experimental datasets to further improve the robustness, generalizability, and statistical reliability of the developed models.

### 3.8 Mechanical interpretation of tensile strength enhancement

While the machine learning models developed have achieved high prediction accuracy, the most important contribution made by the research work is that it can generate meaningful information on how the tensile strength can be increased in FWTPET joints. The application of physics inspired engineering features together with Explainable Artificial Intelligence (XAI) allows the researchers to provide prediction results that are related to thermo-mechanical processes occurring during welding. Rather than being a black-box predictor only, the proposed model can pinpoint the key process factors and engineered characteristics that improve joint strength. The SHAP analysis indicates that CNT content, Dispersion Index, Composite Interaction, Composite Ratio, rotational speed, and all other engineering factors are among the most impactful process characteristics in relation to tensile strength. These observations are in line with known metallurgical principles of friction welding processes.

#### 3.8.1 Heat generation and thermo-mechanical coupling

Heat generation is one of the essential elements affecting the quality of friction welding. Friction between the rotating tool and the workpiece during the FWTPET process converts mechanical energy into heat and allows for heat-induced softening without melting the base material. The authors proposed three physics-based measures: namely, the Heat Generation Index (RPM × Depth), Specific Energy Index (RPM/Depth), and Composite Heat Index ((RPM × Depth) × (CNT + Si₃N₄)), which aim to introduce physics-based concepts for describing the observed behaviors.

When tool speed increases, so does the amount of friction work done at the tool-workpiece interface resulting in local temperature in the stir zone. The same principle applies to plunge depth increased depth means higher pressure on the contact surface, leading to greater plastic work and, consequently, higher heat generation. Additionally, the Composite Heat Index also considers reinforcement loading and, thus, indicates that the effectiveness of heating depends not only on processing parameters but also on the amount of the reinforcing phase present in the weld zone.

The appropriate amount of heat production lowers the flow stress in the aluminum matrix, which supports plastic deformation and the full consolidation of the tube-to-plate interface. On the other hand, insufficient heat means that bonding is incomplete, the material flow is bad, and adhesion between the materials is weak. Excessive heat generation can cause matrix softening, grain growth, and instability in the stirred area. Thus, machine learning has successfully determined that the heat parameters are crucial because they directly influence the thermo-mechanical properties essential for producing high-quality welds.

#### 3.8.2 Material flow and mixing efficiency

Material flow is another critical factor influencing the mechanical properties of friction welded components. The deformation generated by the rotating elements results into softened aluminum movement around pin of the tool while the reinforcing particles are dispersed in the stirred zone. The Mixing Index value (RPM x (CNT + Si₃N₄)) is said to present the effects of both mixing process intensity and concentration of reinforcement. As the rotation speed increases, so does the momentum of rotation and shear deformation, enabling better distribution of CNTs and Si₃N₄ within the weld nugget. Equal distribution of reinforcement particles means to minimize localized agglomeration of reinforcements. The correlation analysis indicated that the factors associated with mixing greatly affect the strength of the joint.

#### 3.8.3 Plastic deformation and consolidation

Plastic deformation is at the heart of metallurgical bonding in the friction stir welding process. The downward force from the spinning tool applies high plastic deformation in the aluminum, leading to excellent contact between the pipe and the pipe plate while removing the gap that exists between the two in the interface. The plunge depth mostly dictates the amount of deformation experienced by the material. Plunge depth is also necessary to determine the size of the material being deformed as when the plunge depth increases, more material undergoes compressive forging, thus creating better conditions for consolidation and interfacial closure. When the plunge depth is in line with the correct rotational speed, the deformation contributes to dynamic flow of the material and complete bonding in the weld.

Described engineered parameters for plunge depth also reveal this deformation aspect as they explore the interconnected effects of the rotational speed, heat produced and plastic work. The improvement in consolidation results in lowering the internal discontinuities thus enhancing the load-bearing capability of the pipe joint and therefore its tensile strength.

#### 3.8.4 Reinforcement dispersion mechanisms

The use of CNT and Si₃N₄ particles provides one of the most important benefits of creating a hybrid reinforcement system that enhances both strength and structural stability. The effectiveness of these reinforcements, however, is strongly dependent on their dispersion in the stirred material. The Dispersion Index (RPM × CNT) quantifies the rotational contribution to CNT dispersion, and the Ceramic Dispersion Index (RPM × Si₃N₄) quantifies the effectiveness of stirring in ceramic particle dispersion. The synergistic interaction between the two reinforcement phases is also captured in the Composite Interaction (CNT × Si₃N₄).

To separate bundled nanotubes and reduce agglomeration, the carbon nanotubes need sufficient shear deformation. After uniform dispersion, CNTs fill in the microcracks, transfer the stress, and increase the tensile strength and ductility. Si₃N₄ particles, on the other hand, are mainly responsible for its hardness, wear resistance and resistance to local plastic deformations. The combination of these complementary strengthening mechanisms, namely crack-bridging, and particle reinforcement, makes the hybrid interaction between CNT and Si₃N₄.

The XAI analysis results indicate that the Dispersion Index and Composite Interaction are indeed significant, and the reinforcement efficiency is therefore not only dependent on the quantity of reinforcement but also on its distribution. This result agrees with the widely reported composite strengthening mechanisms in the literature of Friction Stir Processing (FSP).

#### 3.8.5 Grain refinement and microstructural strengthening

The use of substantial plastic deformation together with controlled heat treatment of the FWTPET leads to microstructural transformations in the stirred zone. The grain size is not calculated but the developed engineered properties are sensitive enough to the thermo-mechanical conditions that lead to dynamic recrystallization and grain size reduction. The synergies between controlled heat supply and significant plastic deformation make it possible to have small equiaxed grains obtained while uniformly distributed reinforcing particles limit the grain growth, preventing it from moving. CNTs and Si₃N₄ act as dislocation motion barriers, resulting in better resistance to plastic deformation under tension conditions.

The final increase in tensile strength is caused by the refinement of metal structures.

#### 3.8.6 Integrated mechanism of tensile strength enhancement

The increased tensile strength obtained from the developed FWTPET joints are attributed to a joint with a combination of process parameters, thermo-mechanical conditions, reinforcement behavior and microstructural evolution. First, the appropriate rotational speed and plunge depth are selected to generate frictional heat and the plastic deformation in the work piece. These conditions then determine the flow of materials, causing the distribution of CNT and Si₃N₄ particles become uniform in the stirring region.

The engineered descriptors created in this research effectively capture each of these physical processes. Thermal softening and deformation characteristics are called heat-related features, the characteristics of reinforcement transport are called mixing descriptors, and the efficiency of particle distribution is called dispersion descriptors. These mechanisms provide better consolidation, better metallurgical bonding, finer microstructure, and efficient load transfer through the weld.

The Explainable Artificial Intelligence analysis also determined that the most important variables for tensile strength are CNT content and Dispersion Index followed by Composite Interaction and Composite Ratio. The results presented in this work show that the tensile strength is not only dependent on the individual process parameters, but also on the quality of the distribution of the reinforcement and thermo-mechanical treatment conditions. Thus, the proposed physics inspired machine learning method successfully identifies the same governing mechanisms that are traditionally identified from physics-based friction welding metallurgy, which is a verification of the predictive capability and physics interpretability of the developed model.

The mechanical interpretation reveals the developed physics inspired machine learning framework is not limited to data-driven predictions, but it also provides a direct linkage between the process parameters, engineered features, metallurgical phenomena and tensile strength enhancement. The variables with the most influencing effects indicated by the SHAP analysis were CNT content and Dispersion Index, which clearly indicate that joint performance is impacted most significantly by the distribution of reinforcements. Thermal input descriptors such as the Heat Generation Index, Specific Energy Index and the Composite Heat Index validated that providing proper thermal energy is critical to achieving proper material softening, plastic deformation, and defect-free consolidation. Similarly, the Mixing Index, Composite Interaction and reinforcement dispersion descriptors highlighted the need for uniformity of particle distribution for good load transfer and crack resistance.

These thermo-mechanical mechanisms help to realize better consolidation, fine grains, better interlayer bonding, and effective transfer of stress through the welding interface, achieving better tensile strength at the welded interface. The XAI results confirmed with existing friction welding metallurgy results show that the proposed framework is not a predictive model but also a scientifically interpretable tool that can explain the physical mechanisms that influence the performance of the CNT–Si₃N₄ reinforced friction welding process. This work combines the fields of Mechanical Engineering and Explainable Artificial Intelligence, contributing greatly to the creation of trustworthy, explainable, and physics inspired machine learning models for advanced manufacturing.

## 4 Limitations of the research

The present research proves the ability of physics inspired machine learning to predict the tensile strength of the CNT–Si₃N₄ reinforced FWTPET joints. Several constraints are, however, recognized. First, the developed models were trained with a relatively limited experimental database of 25 observations, which is limited mostly because of the fabrication and mechanical testing costs. To make better use of the data, LOOCV was used, but with larger experimental datasets, the models would be more robust and statistically reliable. Second, only internal cross-validation of the predictive models was performed and there was no independent external validation set. Therefore, one should interpret the reported predictive performance in light of the available experimental data. Third, although stringent validation procedures were carried out, the likelihood of optimistic performance estimates, based on a limited data set, must not be overlooked. Fourth, engineered descriptors have significant multicollinearity due to the mathematical nature of the obtained descriptors as they are related to the same experimental process parameters. Despite being less sensitive to multicollinearity, tree-based machine learning algorithms can be used to gain insight into the relationship between features when the model is considered as part of the interpretation. Lastly, the proposed framework was designed for the investigated CNT–Si₃N₄ reinforced FWTPET process, and further experimental testing is needed to confirm its applicability to other materials, reinforcement systems, welding configurations and/or processing conditions. Future studies will be aimed at increasing the experimental database, the use of independent validation databases, the use of nested cross validation strategies, and testing the proposed methodology on a wider variety of friction based joining applications.

## 5 Conclusion

This research developed a machine learning framework for tensile strength prediction of CNT–Si₃N₄ reinforced FWTPET joints based on 25 experimentally obtained observations. The proposed method incorporates physics-inspired engineered descriptors along with traditional process variables to offer another representation of the interaction of thermal, mechanical and reinforcement forces during welding. From the process parameters measured from the experiments, descriptors related to heat generation, material flow, reinforcement dispersion, composite interaction, and thermo-mechanical coupling were formulated and then statistically analyzed and feature-selection applied.

The six machine learning regression models, XGBoost, GPR, Random Forest, CatBoost, SVR and LightGBM, were tested with LOOCV. To find out the appropriate configurations of the model, hyperparameter optimization was used, which is based on the Optuna optimization framework. XGBoost model shows the highest predictive performance with the lowest MAE of 5.70, RMSE of 7.74, R^2^ of 0.9772, adjusted R^2^ of 0.9608 and MAPE of 1.26%, among the evaluated models. It is observed that the tensile strength has strong predictive information in the experimental process parameters within the process window under investigation.

The feature analysis was used to gain further insight into the variables used by the machine-learning models. The most significant and consistently found feature was the CNT, followed by the Dispersion Index, the Composite Interaction and the Composite Ratio. The physics-derived descriptors were thus found to be convenient representations to understand the relationships among reinforcement content, rotational processing conditions and tensile strength. SHAP analysis further enabled the contribution of individual features to the model predictions to be examined. The results and their mechanical interpretations should not be interpreted as evidence of physical causal mechanisms but as links made by the model that refer to the principles of welding and materials.

Ablation analysis was a valuable assessment of the true contribution of the engineered features. The model with the original experimental variables (CNT, Si₃N₄, RPM and Depth) gave better predictive performance, than the models with the physics-inspired descriptors only and the combined feature set for the present data. Therefore, the results are not indicative of the superiority of the engineered descriptors when it comes to the predictive accuracy when compared with the original variables. Their main utility, however, is in offering physically motivated and interpretable representations of the process relationships that supplement the original experimental parameters. This distinction focuses on the scientific validity of the proposed framework and prevents the exaggeration of the contribution of feature engineering in the process of making predictions.

Thermo-mechanical conditions related to the formation of the FWTPET joints can be linked to the mechanical aspects of the identified features. The rotational speed and plunge depth affect frictional heating, plastic deformation and material flow; descriptors related to reinforcement represent interaction and distribution of CNT and Si₃N₄ in the processed area. These factors may affect the degree of consolidation, effectiveness of reinforcement, bonding between the two, load transfer, and the tensile response. The SHAP results and the machine learning results are therefore a data-driven perspective, which is interpretable and consistent with the mechanical behavior of reinforced friction-welded joints as expected, but not a statement of the physical mechanisms.

However, a few limitations should be noted, although the predictions were encouraging. Only 25 experimental observations were used to develop the framework and there was no independent external data set to validate the framework. Moreover, feature selection and hyperparameter optimization were performed outside of the nested procedures in each LOOCV fold, so some optimistic bias in the reported performance remains. There is also significant multicollinearity among the engineered descriptors, as they are mathematically related to each other and to the original process variables. Moreover, the feasibility of the proposed model for other processing conditions of CNT–Si₃N₄, AA6061/AA6063 and FWTPET needs to be validated.

Overall, the research illustrated the feasibility of incorporating small-data machine learning, physics-inspired feature engineering and explainable artificial intelligence in the analysis of the tensile strength of friction-welded composite joints. The results offer a framework of interpretation and analysis of experimental obtained welding data which is efficient in terms of the data, while clearly distinguishing between predictive modeling and physical causality. Additional experiments are suggested to increase the experimental database, perform independent experiments to validate the framework and include fully nested cross-validation to determine the generalizability and practical applicability of the framework across a wider material composition and processing conditions. Although the predictive performance in the present research looks promising, one needs to validate the findings based on larger experimental dataset and cross-validation process to improve the generalizability of the proposed model and its statistical power.

## Data Availability

The data supporting the findings of this study are available from the corresponding author upon reasonable request. The data from the previous study used in the present research are available as reported in Siddhardh et al. (2020).
